# Metagenomics Insights Into the Microbial Diversity and Microbiome Network Analysis on the Heterogeneity of Influent to Effluent Water

**DOI:** 10.3389/fmicb.2022.779196

**Published:** 2022-04-14

**Authors:** Bahiyah Azli, Mohd Nasharudin Razak, Abdul Rahman Omar, Nor Azimah Mohd Zain, Fatimah Abdul Razak, I. Nurulfiza

**Affiliations:** ^1^Laboratory of Vaccines and Biomolecules, Institute of Bioscience, Universiti Putra Malaysia, Seri Kembangan, Malaysia; ^2^Department of Cell and Molecular Biology, Faculty of Biotechnology and Biomolecular Sciences, Universiti Putra Malaysia, Seri Kembangan, Malaysia; ^3^Faculty of Veterinary Medicine, Universiti Putra Malaysia, Seri Kembangan, Malaysia; ^4^Department of Biosciences, Faculty of Biosciences and Medical Engineering, Universiti Teknologi Malaysia, Skudai, Malaysia; ^5^Research Institute for Sustainable Environment, Universiti Teknologi Malaysia, Skudai, Malaysia; ^6^Department of Mathematical Sciences, Faculty of Science and Technology, Universiti Kebangsaan Malaysia, Bangi, Malaysia

**Keywords:** WWTP (wastewater treatment plant), population, metagenomic, sewage, comprehensive antibiotic resistance database, co-occurrence network, microbial community, antibiotic resistance genes

## Abstract

Sanitizing the water sources of local communities is important to control the spread of microbial resistance genes, especially those for water-borne illnesses. The activities of antibiotic resistance gene (ARG)-host pathogens pose a threat to public health, and it has been estimated that the infection will lead up to 10 million deaths globally by the year 2050. Hence, in this study, we aim to analyze the efficiency of our municipal wastewater treatment plant (WWTP) process in producing pathogen-free water by investigating the microbial composition between influent and effluent water sites. Shotgun metagenomics sequencing using the Illumina platform was performed on the influent and effluent samples of six different WWTP sites located in Johore, Malaysia. After raw data pre-processing, the non-redundant contigs library was then aligned against BLASTP for taxonomy profiling and the Comprehensive Antibiotic Resistance Database for ARG annotation. Interestingly, the alpha-diversity result reported that effluent site samples showed higher abundance and diverse heterogeneity compared to the influent site. The principal component analysis (PCA) and non-metric multidimensional scaling (NMDS) plots also suggested that effluent sites showed high variation in the genetic material due to loosely clustered sample plots, as compared to the tightly clustered influent samples. This study has successfully identified the top three abundant phyla in influent—*Proteobacteria*, *Firmicutes*, and *Bacteroidetes*—and effluent—*Proteobacteria*, *Actinobacteria*, and *Bacteroidetes*—water. Despite the overlap within the top three abundant phyla in influent and effluent sites (*Proteobacteria* and *Bacteroidetes*), the ARG composition heat map and drug class phenotype plot bar exhibits a general trend of a downward shift, showing the efficiency of WWTP in reducing opportunistic pathogens. Overall, it was demonstrated that our municipal WWTP efficiently eliminated pathogenic microbes from the influent water before its total discharge to the environment, though not with the total elimination of microorganisms. This metagenomics study allowed for an examination of our water source and showed the potential interaction of species and ARGs residing in the influent and effluent environment. Both microbial profile structure and co-occurrence network analysis provide integrated understanding regarding the diversity of microorganisms and interactions for future advanced water sanitation treatments.

## Introduction

The safety of our water system and water supply defines the future health and well-being of a nation. Water-borne pathogens are responsible for many types of illnesses, including gastrointestinal illness, neurological illness, skin problems, bloodstream infections, respiratory-related illness, and more. Wastewater treatment plants (WWTPs) are common reservoirs of a variety of pathogens. Domestic wastewater profiling shows a high concentration of microbial and viral contaminants accumulated from various locations within the agriculture, hospital, industry, and household sectors. A well-functioning WWTP is essential to a living community as it is often a reservoir for clinical antibiotic-resistant bacteria with increased resistance to antibiotics. Owing to the opportunistic nature of pathogens, the high diversity of genetic material in WWTPs encourages their vertical or horizontal transmission between microorganisms to increase their survival capability (Osińska et al., 2019). The antibiotic-resistant bacteria can transfer their antibiotic resistance genes (ARGs) to human pathogens and thus increase the prevalence of infection, especially *via* the fecal–oral route (Osińska et al., 2019). Among microbial pathogens, bacteria are a chief concern as they can grow and replicate independently without a host cell (Al-Gheethi et al., 2018). According to the World Health Organization (WHO)’s One Health policy, analyzing the WWTP sources of nearby local communities is important to ensure the public is continuously provided with a clean environment (O’Brien and Xagoraraki, 2019). Interdisciplinary approaches are needed in all areas of the community to control this issue, ranging from prevention, education, investments, and policy development. Efficient technology is a necessary measure of protection against pathogens, specifically to prevent them from growing and spreading in drinking water. Any data that shed light upon waterborne disease etiology or pathology will help to mitigate the waterborne disease threat globally.

Microorganism diversity is reported annually, showing that these living organisms evolve rapidly as a result of 3.8 billion years of evolution (Shikha et al., 2021). Culture-independent techniques are needed to explore the genetic diversity, ecological roles, and population structures of uncultured microorganisms that can be employed without the traditional method of isolation and culture due to certain limitations. 16S metagenomics analysis was the first bioinformatics analysis employed to dispense microbial information. Yet, this metagenomics technique is limited to only generating microbial structure profiles, and it shows an inability to retrieve in-depth information of functional roles. Employing shotgun metagenomics is the recommended technique to obtain taxonomy profiling information and related functional roles, such as gene annotation, for a thorough analysis. This study employs the examination of genetic material present in an environment obtained from sampling and of the inherent diversity of microscopic life, allowing fellows experts in this field to conduct large-scale investigations in a shorter time and with high confidence. Various fields have employed this technology as one of the main investigation methods; this is widely practiced in the health, agriculture, and ecology sectors, such as when creating a microbiome profile of the gut flora (Mancabelli et al., 2017; Saito et al., 2019; Xu et al., 2019). The analysis of the profile generated from the metagenomics process would indirectly answer queries regarding the presence of ARGs and suggestions on how to combat public health problems caused by environmental pathogens (Guazzaroni et al., 2009). Hence, the employment of a metagenomics analysis using the shotgun sequencing method suggests future initiatives for pathogen removal.

Furthermore, shotgun metagenomics data can provide beneficial insights into the interaction between and co-occurrence of ARGs in a control population or environment (Chaffron et al., 2010). The co-occurrence ARG network, however, provides the basis for the potential ARG cluster regulation initiatives and future studies. Nevertheless, it is important to note that the final genomic-network interpretation is computationally challenging and would have been limited by factors such as community turnover and local niches of the samples. Numerous studies have applied the metagenomics network approach to find significant biological interactions to generate a hypothesis, especially in tackling the issue of antibiotic resistance of microbial species in the gastrointestinal tract (Zhang et al., 2014), soils (Barberán et al., 2012), and activated sludge (Ju et al., 2014).

Hence, we aim to evaluate the efficiency of Malaysia’s WWTPs in producing drinking water that is safe from the pathogenic microbiome by using the shotgun metagenomics technology. This study aims to do the following: (1) compare the microbial community profiles in the influent and effluent samples from a WWTP; (2) analyze the presence of ARGs in influent and effluent sites; (3) generate a co-occurrence network of influent-only and effluent-only environments.

Proper surveillance and research analysis of employed treatment in WWTPs are required to ensure efficient removal of these pathogenic bacteria before the water is released into a natural body of water (Von Sperling, 2007; Narayanan and Narayan, 2019). In this paper, we show the efficiency of the municipal WWTP performance in reducing the bacterial populations and the ARGs that persisted even after the treatment. The ARG information retrieved was analyzed to predict the co-occurrence interaction among the selected highly pathogenic bacteria responsible for public health concerns and the ARG clusters.

## Materials and Methods

### Sample Collection

**FIGURE 1:**
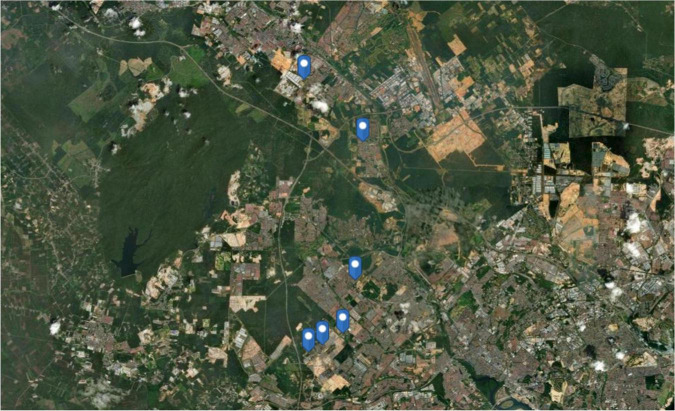
Geographical marking points of six chosen wastewater treatment plants as sampling stations in Johore, Malaysia, created using ArcGIS^®^ software by Esri.

Six different WWTPs (Figure 1) were selected as the sampling locations in this microbial shotgun metagenomics study. The WWTPs included three main removal stages to eliminate the contaminant or polluted materials: the primary, secondary, and tertiary stages (Ward et al., 2008). The samples from the WWTPs were collected on the 6–7 of November 2011 from 8 am to 4 pm. For each WWTP, samples of 1 L of wastewater were collected in three separate sterile flasks. The influent wastewater was taken before the first settling tank, while another 1 L of effluent wastewater was taken after processing at the third plant reactor. To sum up, six flasks in total were collected to represent each WWTP. Thus, 36 collected samples were then stored in a cooler during the collection time. Once the samples arrived at the laboratory, 500 mL of each water sample was stored in 10 Falcon tubes holding 50 mL each. Then, all sample tubes were stored in the freezer at −20°C until further use. Notably, the samples of influent and effluent wastewaters collected were labeled as “I” and “E,” respectively.

### DNA Isolation and Shotgun Sequencing

All 12 previously collected samples were subjected to DNA isolation using the E.Z.N.A^®^ Soil DNA extraction kit (Omega Bio-tek, United States). The performance assay was performed according to the manufacturer’s guidelines. Briefly, 50 ng of samples underwent the fragmentation of ∼300 bp as DNA sample optimization in the M220 focused-ultrasonicator instrument (Covaris, United States).

Total DNA was subjected to DNA libraries construction and amplification using the TruSeq™ DNA Sample Prep Kit (Illumina, United States) and the cBOT TruSeq PE Cluster Kit v3 (Illumina, United States) reagents, following the manufacturer’s guidelines. The DNA libraries were sequenced using the Illumina HiSeq technology with the TruSeq SBS Kit v3-HS (Illumina, United States) set in the 300 cycles environment. The sample DNA libraries were then pooled in an equimolar amount and subjected to a paired-end (2 × 150 bp)-sequenced platform according to the standard Illumina protocols. The raw reads were stored in the fastq format. The number of raw reads produced is presented in Supplementary Table 1. These reads were further subjected to a typical downstream shotgun metagenomics pipeline: (1) pre-processing and (2) clean reads analysis. High-quality DNA sequences libraries used in this study have been deposited in the NCBI Sequence Read Archive (SRA) with the BioProject No. of PRJNA693629.

### Sequences Raw Reads Pre-processing

The produced raw reads outputs were later subjected to processing for quality assessment and quality checking. Linkers sequences from the raw reads were removed. Using the trimmomatic software (Bolger et al., 2014), the linker-free raw reads were then trimmed at the bases with quality <20 at the 3′ end, removed if N content exceeded 10%, and discarded if the sequences were <70 bp in length after quality trimming. To further improve the quality and reliability of the raw reads prior to investigation and analysis, the quality-trimmed reads were performed onto the SeqPrep (GitHub - jstjohn/SeqPrep, 2021: *Tool for stripping adaptors and/or merging paired reads with overlap into single reads*., no date) software for quality control. Next, Sickle (Joshi and Fass, 2011) was applied to remove reads with the length <50 bp, mean quality <20, and those with ambiguous bases (N). The total number of clean reads of all samples following pre-processing is presented in Supplementary Table 2. We decided to further analyze the data as most of the reads were of good quality. To ensure that there is no genomic noise caused by a large number of data or contamination that occurs in the reads, the pre-processed clean reads were further performed onto the host genome removal software Burrows–Wheeler Aligner (BWA) (Li and Durbin, 2010).

### Sequence Assembly, Gene Prediction, and Homolog Gene Clustering

Reads assembly was conducted using the SOAPdenovo v.1.06 software (Li et al., 2008). Clean data with the *k*-mer ranging from 39 to 47 were assembled into the fasta format, and the best assemblies, as shown in Supplementary Table 3, were selected. To maintain the quality of the clean reads, only contigs >500 bp in length were retained for further analysis, while the rest was discarded.

The contigs produced were subjected to gene prediction using the MetaGene software (Noguchi et al., 2006). Only coding regions with the open-reading frame (ORF) in the assembled contigs with the length >60 bp were retained and translated into amino acid sequences. The statistics of metagenomic sequencing and assembly results as well as the predicted ORF information for all samples are displayed in Supplementary Tables 3, 4, respectively.

Finally, all the predicted genes were clustered using the CD-HIT (Cluster Database at High Identity with Tolerance) software (Fu et al., 2012) to produce a non-redundant (NR) gene catalog. The parameter, identity, and coverage were set in the software as 95 and 90%, respectively. As for the results, only the longest genes of each cluster were selected as the representative sequences for the NR gene catalog construction. The removal of redundant sequences in each sample prevented the over-reporting of subsequent results along with the analysis.

### Gene Profile Analysis

Using the ggvenn software (Yan, 2021), a Venn diagram representing the gene profile of influent and effluent samples retrieved from the MetaGene and CD-HIT processing stage was generated. The gene profile size results for all 12 samples obtained from the previous assembly method were treated as the input. To further investigate the metagenomic composition of the two source groups (influent and effluent), UpSet plots were produced from the UpSet R (Conway et al., 2017), using the gene profile size of samples as an input, according to their respective group.

### Reads-Based Taxonomy Profiling

To retrieve the identity of each sequence, the NR gene catalog was aligned with NR databases of NCBI using the BLASTP v.2.2.28+ (Altschul, 1997). Only hits with a cut-off *e*-value < 1e−5 were used. The NR database of NCBI includes the Swiss-Prot, Protein Information Resources, Protein Research Foundation, and Protein Data Bank. Meanwhile, the translated protein sequences information was from the GenBank and RefSeq databases. Taxonomy profiling of the clean reads was conducted using the Kraken-miniDB database (Wood and Salzberg, 2014) by the Kraken analysis. According to the results, taxon abundances were assigned with each taxonomic level such as domain, kingdom, phylum, class, order, family, genus, and species. Only the domain-, phylum-, class-, order-, family-, genus-, and species-level information were selected for a thorough analysis in the study.

### Species Composition Analysis

Overall composition profile of the NR gene catalog taxonomy was analyzed using alpha-diversity metrics, specifically the richness, abundance, Shannon (H), and Simpson 1-D index. The full gene taxonomy profile obtained from the taxonomy (Supplementary Table 6) binning was used as the input for the alpha-diversity metrics analysis and performed onto the Vegan R (Oksanen et al., 2008).

To analyze the contrast between the abundance of the microbial population of the influent and effluent samples, bar plots representing the phylum-, class-, order-, family-, and genus-level results were generated using the built-in functions of R. Among 192 phylum-taxa (Supplementary Table 7), 353 class-taxa (Supplementary Table 8), 617 order-taxa (Supplementary Table 9), 1089 family-taxa (Supplementary Table 10), and 3435 genus-taxa (Supplementary Table 11), only column sums of relative abundance value > 1% were retained, while the discarded ones were newly classified under “Other.” Furthermore, microorganisms with a percentage <1% were labeled as “Other.” Each taxon in all levels is represented by different colors.

Next, to evaluate the microbial community structure between samples, principal component analysis (PCA) and non-multidimensional scaling (NMDS) were generated. The PAleontological STatistics (PAST) v.4.03 (Hammer et al., 2001) was used to produce the ordination plots, in which each source was represented by specific icons with the Bray–Curtis set as the matrix distance. The phylum and genus composition profiles were used as the input to explore the relationship of bacterial community structure in the wastewater samples. Relative loading values of PCA (Supplementary Table 13) and NMDS (Supplementary Table 14) used to build the ordination plots are reported for future reproducibility. Using PAST software, the similarity percentage breakdown (SIMPER) calculation option was also performed onto the same data used for PCA and NMDS, for further interpretation of the two ordinations plot and structure.

### Antibiotic Resistance Gene Functional Abundance Profiling

To analyze the abundance of antibiotic resistance function of the shotgun metagenomics samples, an analysis of the Comprehensive Antibiotic Resistance Database (CARD) ARG was carried out *via* the in-built website Resistance Gene Identifier tool (Alcock et al., 2019). This database contains a wide range of reference genes related to antibiotic resistance from a variety of organisms, genomes, and plasmids. In addition, Antibiotic Resistance Ontology (ARO) is the core database of CARD, and it provides a functional classification for the CARD analysis. Reads were annotated using the best hit to the database, with a 95% identity and 95% alignment coverage set as the query parameters. The taxonomy information of the best hit in the NR database was used to predict the taxonomy of each gene, and the relative abundance of each taxonomic level was counted by summing the abundance of each gene belonging to it. Only perfect and strict hits against the database will be retained in the analysis, such as an *e*-value < 1. All antibiotic resistance annotations produced from the analysis such as the CARD ID, name, ARG family, drug class phenotype, and resistance mechanisms are displayed in Supplementary Table 15.

Box plots of the antibiotic resistance type abundancy profile (Supplementary Table 16) and drug class profile (Supplementary Table 17) for each sample were produced and displayed in Figures 8, 9 to analyze the phenotypic profile of ARG. The samples were grouped considering respective sources to observe the differences in phenotype. Next, an overview displaying an annotated heat map of ARGs with a sum abundance of >0.1% was generated to compare wastewater samples using the relative abundance of ARG values as an input.

Then, the resistomes from eight ARGS families—blaTEM, blaSHV, blaCTX-M, blaOXA, Tet, Sul, Cat, and MDF types—were selected to be discussed in-depth in this study, as these listed ARG families were few of the most concerning pathogens for human health. Retrieved values were plotted into a heat map in R.

### Co-occurrence Gene Network Construction and Analysis

To identify potential ARG-host interaction of 8 selected ARGs, a gene co-occurrence network was constructed using 12 samples from the 2 water sources. The correlation matrix between the ARG pairs of influent and effluent samples was statistically calculated using pairwise a Spearman’s correlation rank test (Spearman, 1904) with the Vegan R package (Oksanen et al., 2008). The value produced by the matrix represented the co-occurrence correlation of the ARGs in which a positive matrix indicated a positive correlation, while a negative value indicated a negative interaction. The matrix values were input into Gephi^®^ (Bastian et al., 2009) to plot the gene network, using built-in functions. The generated network consisted of the ARG–ARG, ARG–species, and species–species interactions with the Force Atlas 2 layout format.

To produce a good network, only statistically significant values of *p*-value < 0.01 with a correlation coefficient >0.8 were plotted in the constructed network for an easier interpretation (Barberán et al., 2012; Mandakovic et al., 2018; Yasir, 2020). Meanwhile, the node size was scaled based on the abundance of ARGs in the metagenomics data. Furthermore, using the constructed network, the topological properties of the co-occurrence gene meta-network information are listed in Supplementary Table 19.

### Statistical Analysis

A non-parametric Wilcoxon rank-sum test (Wilcoxon, 1945) was used to analyze the difference between the microbial community and functional diversity using the IBM SPSS Statistics 26.0 (IBM, 2019), Vegan (Oksanen et al., 2008), ggbiplot (Wickham, 2016), and indicspecies R (De Caceres and Legendre, 2009), specifically in the analysis of the domain, phylum, class, order, family, genus, resistance mechanism, and drug class profile. Meanwhile, analysis of similarities (ANOSIM) statistical tests (Clarke and Warwick, 1994) were employed on the ordination plots. In brief, any *p*-value < 0.05 was statically significant. Finally, Spearman’s correlation rank test (Spearman, 1904) was performed in the R environment (R Core Team, 2021) to retrieve the matrices value for co-occurrence meta-network generation.

## Results

### Metagenome Sequencing Overview

The PCR amplification and Illumina sequencing step performed produced high-quality metagenomics libraries for all 12 samples. Hence, high-quality contig sequences were retrieved successfully for the analysis, approximately within the range of 110,539–260,732 contigs with the bp lengths around 194,040,642 and 35,362,274, respectively (Supplementary Table 3). During the ORFs retrieval during the gene prediction step, a range from 295,038 to 549,163 ORFs with a total length of 180,456,013 and 288,008,513 bp were reported (Supplementary Table 4). This gene profile information is deemed appropriate for the investigation of microbial profiles between influent and effluent water sources.

**FIGURE 2:**
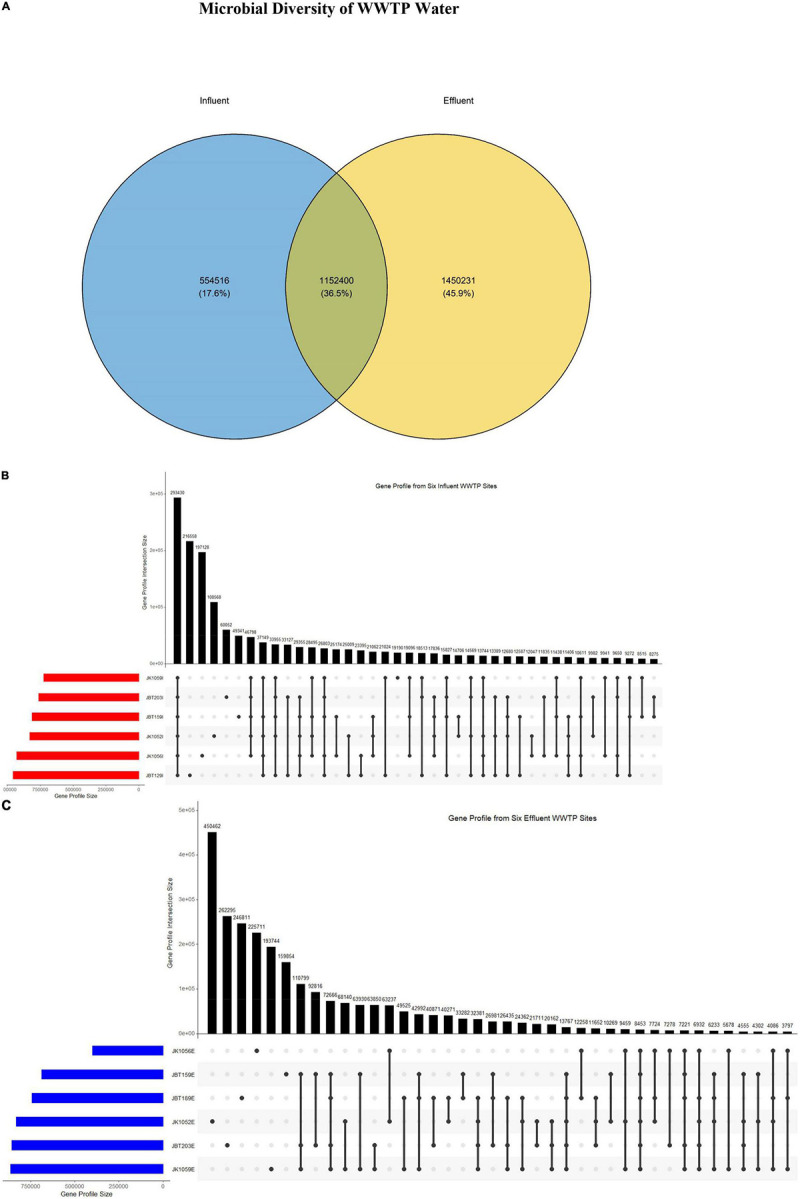
**(A)** Venn diagram consisting of two sets of groups: influent (blue) and effluent (yellow). The UpSet R plots of the gene profile from **(B)** six influent and **(C)** six effluent WWTP samples.

Gene profiles retrieved from the NR gene catalog construction were analyzed to observe overlapping similarities in genes composition between samples. A total of 1,152,400 (36.5%) sequences were reported similar between these two groups. Interestingly, the number of sequences identified in effluent samples only was 45.9% larger compared to the influent samples at 17.6%, approximately 1,450,231 vs. 554,516, respectively (Figure 2A). Accordingly, the number of sequences reported to be uniquely present in the influent-only group was proposed as the size of sequences of pathogenic bacteria, eukaryotes, or viruses that will be eliminated upon treatment. Next, it is observed only influent samples exhibited the largest gene profile size (293,450) as compared to effluent samples gene profile size (8453) (Figures 2B,C), suggesting that the genomic composition of influent water samples is more homogenous within groups, while effluent samples may have large heterogeneity in the genomic composition.

### Alpha-Diversity

**FIGURE 3:**
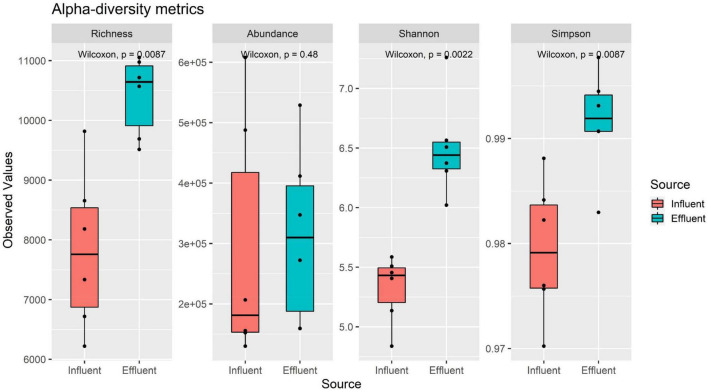
Alpha-diversity metrics of six influent and six effluent samples.

The alpha-diversity analysis was performed to examine the diversity between the influent and effluent samples using the richness, abundance, Shannon, and Simpson diversity indices. The observed values from all four alpha-diversity indices indirectly reported the environment’s community heterogeneity structure. All four diversity indices reported a larger value of mean in the effluent group as compared to the influent group (Figure 3). The observed results suggest that the effluent genomic composition is richer in species, and larger in the abundance value with infinite diversity, as compared to the influent source. This result aligns with findings retrieved from gene profile analysis as the effluent profile suggested high heterogeneity in sequences as compared to the influent profile.

### Taxonomic Composition Profiling

Only the mean differences between the two sources of archaea, bacteria, and “Other” were reported to be statistically significant, while the domains of the eukaryotes and viruses did not show statistically significant differences between the means of sources (Table 1). Interestingly, only bacteria and “Other” were observed to have a negative mean difference, suggesting that only these two domains were reduced successfully in the bioreactor. Meanwhile, eukaryotes, archaea, and viruses showed positive values, indicating an increase of abundance profile in the effluent source. Approximately, 52.4% of the bacteria domain coming from the influent source was eliminated after being treated in the WWTP. This elucidates the efficiency of wastewater treatment to eliminate pathogenic bacteria in outsourced water. The analysis of this domain is the first step in understanding the efficiency of the employed system in WWTPs, specifically toward the bacteria domain.

**TABLE 1:** Tabulated information of domains grouped into respective sources, consisting of the number of samples per group (*N*), mean ± standard deviation (SD), and the mean difference calculated.

Domain	Source	*N*	Mean ± SD	Mean difference	*p*-Value
Archaea	Influent	6	46.09 ± 17.63	517.24	0.002*
	Effluent	6	563.33 ± 109.84		
Bacteria	Influent	6	903371.98 ± 21822.51	−476423.38	0.002*
	Effluent	6	426948.59 ± 136065.36		
Eukaryota	Influent	6	750.20 ± 222.59	2154.99	0.937
	Effluent	6	2905.18 ± 4792.02		
Other	Influent	6	374.71 ± 193.71	−288.97	0.015*
	Effluent	6	85.74 ± 87.22		
Viruses	Influent	6	3837.33 ± 4651.39	6855.88	0.093
	Effluent	6	10693.21 ± 7709.28		

The symbol “*”indicates a p-value < 0.05.

Bar plots were generated to figuratively elucidate the pattern of microbial taxa in influent and effluent water. According to Wilcoxon rank-sum statistical tests, the mean difference of relative abundance of phyla, class, order, family, and genus communities between the two sources showed strong statistically significant differences of 0.0021, 0.0067, 0.0049, 0.0046, and 0.0064, respectively. This confirms that WWTP eliminates bacterial community abundance with significant efficiency in all taxa levels.

**FIGURE 4:**
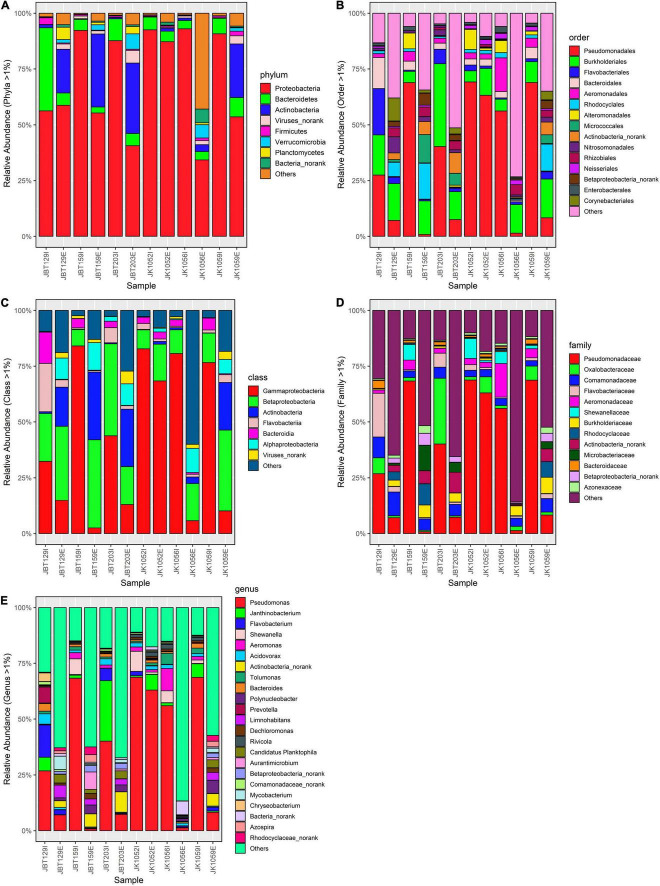
Bar plot of relative **(A)** phyla-taxa, **(B)** class-taxa, **(C)** order-taxa, **(D)** family-taxa, and **(E)** genus-taxa abundance in six influent and six effluent WWTP samples. All taxa levels were reported with *p*-value < 0.05 (Wilcoxon rank-sum statistical test).

In Figure 4A, *Proteobacteria*, *Bacteroidetes*, and *Firmicutes* were the dominant phyla observed in the influent samples, while *Proteobacteria*, *Actinobacteria*, and *Bacteroidetes* were the top three in the effluent samples. Intriguingly, only *Proteobacteria* (67%), *Bacteroidetes* (78%), and *Firmicutes* (58%) phyla showed a downward trend from influent to effluent composition (Supplementary Table 7).

In class-level bar plot (Figure 4B), only *Gammaproteobacteria* (84%), *Betaproteobacteria* (19%), *Flavobacteria* (84%), and *Bacteroidia* (92%) showed reduction from influent to effluent (Supplementary Table 8). Only three out of eight tested class-level taxa produce a *p*-value > 0.05. Meanwhile, in the order-level (Figure 4C), *Pseudomonadales* (85%), *Burkholderiales* (46%), *Bacteroidales* (84%), *Aeromonadales* (93%), *Alteromonadales* (95%), *Rhodocyclales* (94%), *Enterobacteriales* (87%), and *Neisseriales* (59%) were reduced (Supplementary Table 9), with 11 order-level taxa show statistically significant mean difference between the two sources. As for the microbial composition in the family-level (Figure 4D), only 9 taxa were reported with *p*-value < 0.05, with 7 out of 14 filtered taxa showing efficient elimination from influent to effluent, specifically *Pseudomonadaceae* (85%), *Oxalobacteraceae* (86%), *Flavobacteriaceae* (88%), *Aeromonadaceae* (95%), *Comamonadaceae* (35%), *Shewnellaceae* (97%), and *Bacteroidaceae* (90%) (Supplementary Table 10).

The same community profile variation was observed in the genus-level bacterial community analysis. The top three genera with the highest prevalence in the influent source were *Pseudomonas*, Others, and *Janthinobacterium*, while Others, *Pseudomonas*, and *Actinobacteria_norank* were the top three reported in the effluent source (Figure 4E). Among 24 genera, only 19 genera taxa shifted into lower concentration from an influent to an effluent source; *Shewanella* (97%), *Prevotella* (97%), *Chryseobacterium* (96%) *Aeromonas* (95%), *Tolumonas* (93%), and *Bacteroidetes* (90%) were the genera with greatest differences larger than 90% (Supplementary Table 11). Despite being reported as statistically significant in producing the mean difference of all analyzed taxa levels, further elucidation is necessary before concluding the effectiveness of the treatment employed to eliminate bacterial pathogens in the filtering system specifically for investigating the potential of pathogen-activity risk presence in both water sources.

### Ordination

**FIGURE 5:**
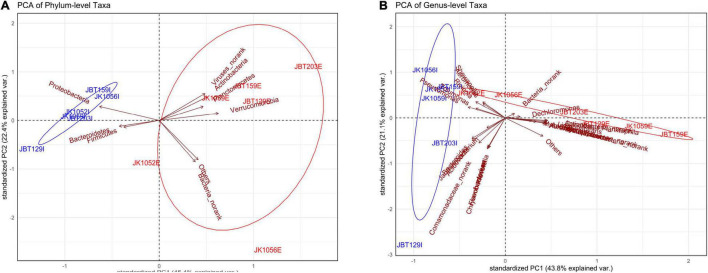
Principal component analysis (PCA) of the **(A)** phylum-level and **(B)** genus-level microbial taxa composition.

Once the global pattern of microbial phyla and genus taxa were observed, we further analyzed the difference in the composition of the samples. Both ordination plots were generated using the phylum taxa and genus input from the NR catalog. As displayed in Figure 5A, samples from the influent water were seen clustered abundantly in a tight community as compared to effluent samples, which are plotted sporadically and in a large dispersal manner. It can be seen that *Proteobacteria*, *Bacteroidetes*, and *Firmicutes* play a large role in contributing to the principal component of the influent samples compared to contributing toward effluent sites (Supplementary Table 13). Meanwhile, there are overlapping of variables of contributors seen in Figure 5B, such as *Pseudomonas*, *Tolumonas*, *Shewanella*, and *Aeromonas*, which indicates that several taxa are highly correlated (Supplementary Table 14). However, these mentioned genus taxa are interpreted to be contributing toward the influent site composition and JK1052E; these are displayed to have similar properties as the influent group. Similarly, both PCA of phylum- and genus-level elucidates high variation of properties of the effluent group, further indicating of high heterogeneity microbial taxa structure.

**FIGURE 6:**
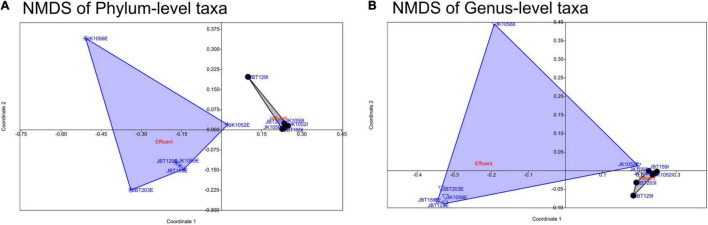
Non-multidimensional scaling of **(A)** phylum-level (ANOSIM statistical test of the Bray–Curtis similarity index, *p*-value = 0.0015) and **(B)** genus-level (ANOSIM statistical test with the Bray–Curtis similarity index, *p*-value = 0.007) microbial taxa composition.

The stress-value of both NMDS plots generated show a *p*-value < 0.1, elucidating differences between the samples for both the phyla (stress-value = 0.0707) and genus levels (stress-value = 0.09741) compositions. The stress-value indicates the generated NMDS ordination space is fair and has a good representation of objects. As shown in the NMDS plot of the phyla (Figure 6A) and genus levels (Figure 6B), influent samples were clustered closer as compared to the effluent samples, which were dispersed widely along with the ordination value. Regardless of slight variance between the phylum- and genus-level profiles of NMDS, there was a general trend of influent samples composition behaving similarly, while effluent samples displayed a looser community structure. According to SIMPER multivariate analysis, measurement of PAST v.403 (with Bray–Curtis dissimilarity) conducted, phyla- and genus-level ordination plots structure were highly contributed by *Proteobacteria* and *Pseudomonas* with an average contribution of 69.32 and 47.63% (Figures 6A,B, respectively). These mentioned taxa are likely to be the major contributors to any difference seen between influent and effluent sites with respect to each level.

### Antibiotic Resistance Gene Phenotype Profile

**FIGURE 7:**
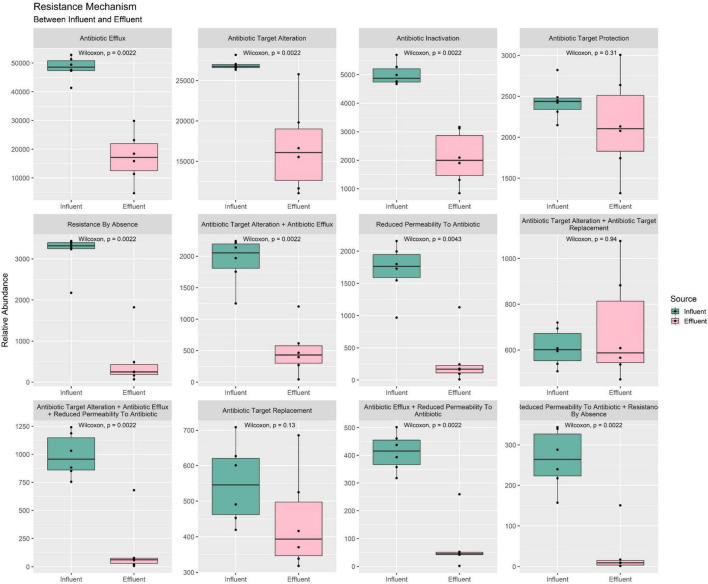
Box plots of the ARG resistance mechanism phenotype between influent and effluent samples.

The CARD database enables the retrieval of ARG and its phenotype information such as the resistance mechanism and drug classes annotation. As presented in Figure 7 total of 12 types of resistance mechanisms were identified in our water samples. Among all mechanisms, only three failed to show a statistically significant difference between the mean of influent and effluent—(1) target replacement, (2) target alteration + target replacement combination, and (3) target protection mechanisms—while the rest were reported with a *p*-value < 0.05. The mean value of influent in all mechanisms was higher compared to the mean of effluent. Moreover, the percentage difference between the sources was >10%. The combination of “reduce permeability to antibiotic + by absence” was reported with the greatest reduction (88%), while the target protection mechanism was reported with the smallest reduction of 12% only (Supplementary Table 16). Surprisingly, the combination of “target alteration + target replacement” was calculated at −13%, showing an ARG increase to be a mechanism to move from influent to effluent composition. Overall, the results indirectly indicated that the treatment efficiently reduced ARG before discharging the water into the community. Nevertheless, further analysis of the mechanism combined with an increment of percentage is required, especially regarding the phenotype of the bacterial community used as a biological treatment in the bioreactor.

**FIGURE 8:**
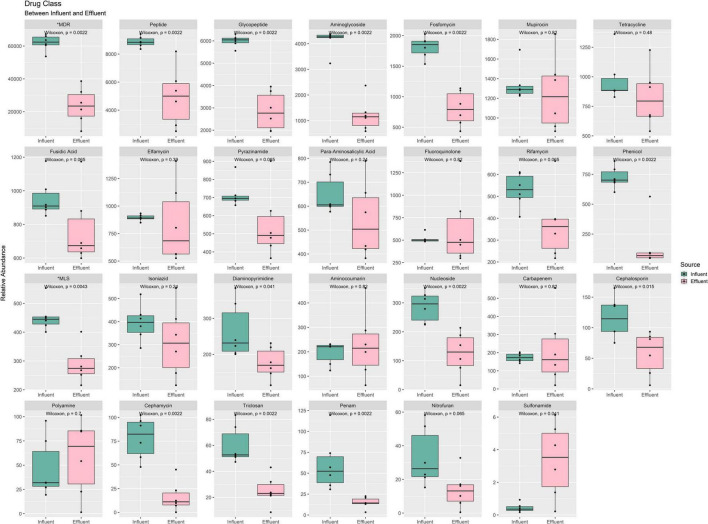
Box plots of the ARG drug class phenotype, grouped between influent and effluent. *MDR, multidrug resistance; *MLS, macrolide–lincosamide–streptogramin.

In Figure 8 total of 27 drug classes were retrieved from the identified ARG in all 12 samples. Only 15 drugs were reported to be statistically significant in the mean difference of influent and effluent. All drug classes show a downward shift from influent to effluent, except for the polyamine and sulfonamide. These drug classes were also observed to have percentage differences ranging from 79 to 7% (Supplementary Table 17). Drug classes with a difference >50% were phenicol (79%), cephamycin (79%), penam (76%), aminoglycoside (70%), MDR (62%), nitrofuran (58%), triclosan (58%), nucleoside (56%), fosfomycin (56%), glycopeptide (52%), and cephalosporin (51%). Only five drug classes were reported with an increase in value from influent to effluent water (aminocoumarin, fluoruinolone, carbapenem, polyamine, and sulfonamide). Interestingly, sulfonamide in the effluent samples shows a drastic increase of six times. Results obtained from analyzing the drug classes phenotype profile of ARG indirectly display the efficient performance of the treatment stages in eliminating pathogenic bacteria that imparted multidrug resistance capacity.

### Antibiotic Resistance Gene Composition Profile

A total of 243 ARG categories, and specifically 1029 ARGs, were initially retrieved upon blasting our NR library into the CARD database (Supplementary Table 18). A total of 582 ARGs were shared among all 12 samples from both sources. Meanwhile, only 65 ARGs were uniquely shared in influent-only and 185 ARGs in effluent-only samples. The shared ARG value difference between influent and effluent samples may elucidate the heterogeneity of pathogen structures between both sources. The difference in the mean of the ARG composition profile between the two sources has been shown to be statistically significant with a Wilcoxon rank-sum test *p*-value of 0.0017. However, only 402 out of 1029 ARGs were observed to have a *p*-value < 0.05. The top five most abundant ARGs were macB, evgS, PvrR, adeL, and tetA(48), whereas macB, tetA(48), cbrA, evgS, and arnA were in the top five observed for the effluent samples. Furthermore, only 64 out of 1029 ARGs were observed to have a downward shift when comparing the mean difference between influent and effluent sources (Supplementary Table 18). Nevertheless, despite having shared the resistomes in both influent and effluent samples, macB (*p*-value = 0.0025), PvrR (*p*-value = 0.0025), evgS (*p*-value = 0.0025), and adeL (*p*-value = 0.0051) have the largest mean difference between two sources, specifically 25, 82, 75, and 78%, suggesting the WWTP filtering system efficiently eliminates ARGs before water discharge.

**FIGURE 9:**
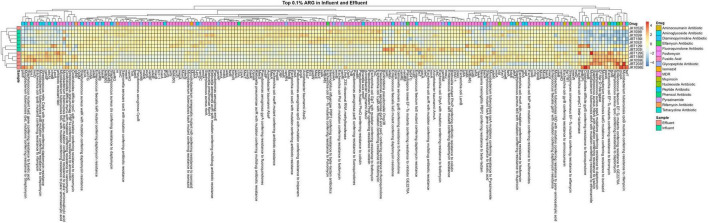
Heat map of the 0.1% ARG in all six influent and six effluent WWTP samples.

For the heat map, only ARGs with a sum row total of 0.1% were selected for the plotting in an explicit analysis. In all 12 samples, a sum of 205 out of 1029 ARGs was selected. In Figure 9, only two major ARG activities were seen in the map, specifically at both ends of the generated heat map. On the right side of the map, several ARG unidentified in the influent sites were reported to be present in the effluent groups. A total of 17 ARGs resistant toward different magnitudes of drug classes were abundant in the treated water, and not in respective influent counterparts. Meanwhile, the left end of the map shows remaining ARGs in effluent water even after being treated in the WWTP, indicating the possibility of incomplete elimination of several pathogens in WWTPs. Further analysis is required to understand and predict the possible factors influencing the increase in resistomes.

**FIGURE 10:**
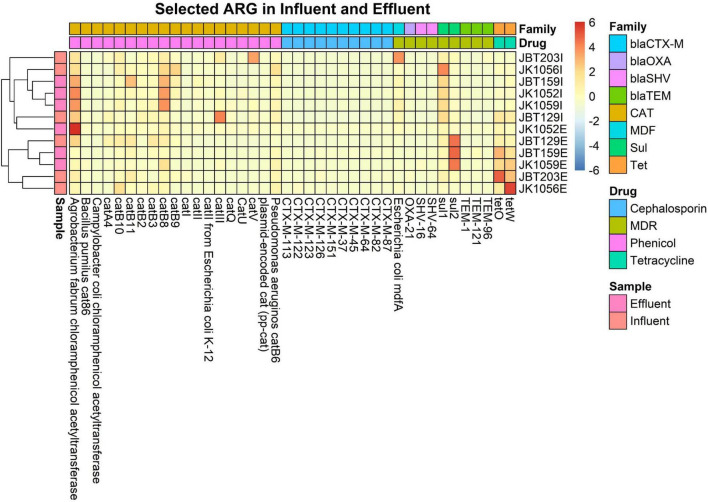
Heat map of 40 selected ARG from 8 interest ARG families, in all 6 influent and 6 effluent WWTP samples.

In total, eight ARG families of interest were further analyzed to observe potential pathogenicity in both sources. From the retrieved CARD output, 40 resistomes were associated with the chosen ARG family of interest. ARGs with high abundance in the influent samples were successfully reduced in the effluent samples, as indicated by the representative color changes from red to blue (Figure 10). Several resistomes with a relatively small abundance profile reported an increase in value in the discharge effluent water. This phenomenon may be caused by the presence of different microbial populations—specifically the pathogenic organisms vs. the bacteria used as bioremediation or favoring the bioreactor’s internal environment.

### Co-occurrence Gene Network

**FIGURE 11:**
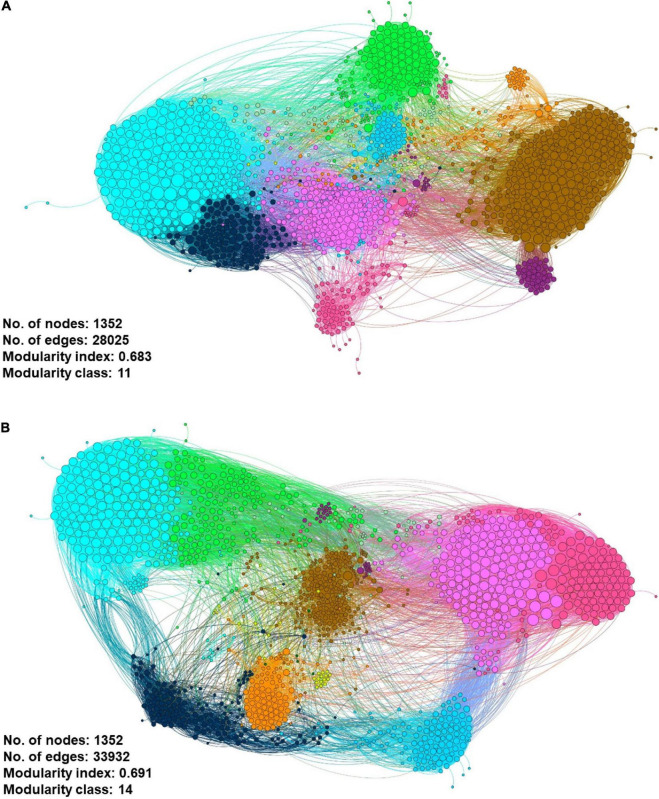
Co-occurrence meta-network analysis showing the correlation between ARG–ARG, ARG–species, and species–species analyzed in **(A)** influent-only and **(B)** effluent-only water environments. Only connections with positive strong Spearman’s ρ rho-value > 0.8 and statistically significant *p*-value FDR-adjusted < 0.01 were displayed in the network. In addition, in the networks, the size of the nodes is proportioned to eigenvector degree, while the width of lines between nodes is proportioned to the correlation coefficient of Spearman’s ρ rho-value. The nodes are colored according to the calculated modularity classes, and they represent either an ARG or species taxa.

We had utilized a correlation-based network approach to explore the non-random co-occurrence patterns of species–species, ARG–species, and ARG–ARG in the municipal WWTP. These meta-networks provide novel information regarding related ARGs and species to our ARG of concern in both water conditions. Only pairwise items of strong Spearman rank’s correlation value (ρ rho-value > 0.8) and significant value (FDR-adjusted *p*-value < 0.01) were retrieved to ensure that only statistically robust interactions were explored. Using the filtered interactions ρ value, gene networks consisting of nodes and edges of ARG–ARG, ARG–species, and species–species were generated *via* Gephi^®^, respective to each source. The co-occurrence meta-network patterns were used to elucidate and propose potential species host of pathogenic ARG. Although both gene networks consist of 1352 nodes, the influent gene network has a smaller edges (interactions) value of 28,025 compared to the effluent gene network of 33,932 (Figure 11). This information may suggest possible differences in the strength of interaction between the nodes (species and/or ARG) in respective sources. Both networks were reported with a modularity index >0.4: 0.683 for the influent and 0.691 for the effluent, respectively. The modularity index values indicate the networks having a concrete modular network structure and not being random, further interpreted as a strong community (i.e., modules, hubs, and clusters) structure (Newman, 2006). As seen in the topography of the influent meta-network (Figure 11A), the ARG and bacterial species were plotted into two big hubs, while the effluent meta-network (Figure 11B) displayed four large and dense hubs. There are 11 and 14 modularity classes displayed in the influent and effluent networks, respectively. These modularity class values represent the groups of microbial taxa that potentially share the same ecological niche without direct interaction. The largest modularity class exhibited in the influent and effluent meta-network is 11 (brown-cluster; 20.86%) and 7 (brown-cluster; 16.35%), respectively. Analysis on the network in the influent sites shows ErmH (*Streptomyces* spp.), Fox1 and Fox5 (*Klebsiella* spp.) and QnrB20 (*Escherichia coli*) nodes as the main hub in class 11, indicating the ARG with the most interaction with other ARGs and species. Meanwhile, there is only one main hub identified in the effluent network generated, which is *E. coli*.

**FIGURE 12:**
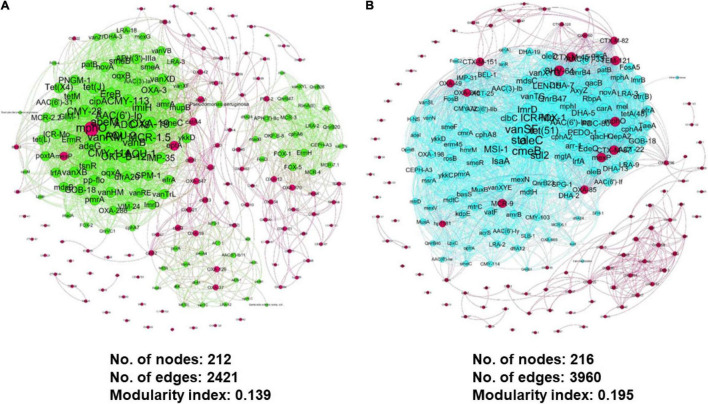
Zoomed and filtered co-occurrence meta-network analysis from the previous global network showing the interaction between selected ARG from eight ARG family types and neighboring nodes in **(A)** influent and **(B)** effluent environment.

Additionally, the meta-network also enables a possible interaction between resistomes of interest and potential pathogenic hosts of concern. The association of clusters in the meta-network can be better illustrated by extracting the selected nodes from the global network and visualizing them separately in a new network. We generated a filtered influent and effluent network (also known as sub-global network) using selected ARG of concern from eight ARG families. *Enterobacteriales* and *Aeromonas sobria* have shown an interaction with the selected ARG in the influent environment (Figure 12A), while *Serratia marcescens* was identified in the effluent environment (Figure 12B). This is a very interesting finding as it may be laid as a foundation for future research in understanding the interaction between microbial population and potential gene transfer for antibiotic resistance-acquired ability in pathogens. Nevertheless, the generated meta-networks that visualize the potential of the microbiome population interacting and co-occurring in favored niches as results for the influent and effluent global meta-networks differ significantly.

## Discussion

All 12 samples of influent and effluent water were collected to investigate the input and output of the wastewater treatment ability to control pathogenic risks and ameliorate environmental health nationwide, regardless of the location. The WWTPs were designed to filter foreign biohazard residuals and pathogenic organisms, both physically and chemically, within a flow of treatment stages such as aerobic activated sludge and anaerobic digestion sludge. At the primary stage, large and bulky objects were blocked from entering the bioreactor to prevent clogging (Archis and Nathanson, 2010). Next, the solid matter undergoes sedimentation at the bottom of the tanks, whilst lighter substances and oil will float. The large sediments were removed and the remaining liquid in the tank was sent to the secondary treatment. Following sedimentation, the water is transferred to the aeration tank for further breakdown at the secondary stage *via* the presence of aerobic bacteria to remove organic components. In some treatment plant systems, a bacteria-growing filter is positioned in the tiles to trap the organic matter when the water passes through it for efficient decomposition. Hence, at this stage, the wastewater is mixed with a small amount of sludge with a high level of oxygen concentration to promote the growth of aerobic bacteria that will remove or consume organic impurities. At the tertiary stage, several methods can be used to further disinfect sewage beyond the primary and secondary treatments. However, the third stage also involves the process of removing the remaining high levels of nitrogen and phosphorous using anaerobic bacteria. To ensure the effluent water is finally safe and drinkable, special equipment and a chemical system of disinfection automation instrument are used to sanitize the water effectively before releasing it into the environment (Ward et al., 2008).

In this study, the gene profile and alpha-diversity analysis reported that the effluent samples were represented with a bigger gene profile size, diverse heterogeneity, higher taxa abundance, and species richness compared to the influent water samples. The findings show the potential of non-pathogenic microbial composition to increase and grow its way to be observed as the dominant taxon in the effluent source. Our ordination plots further supported the observation of high variation in the genetic material seen in effluent sites. Previous microbial diversity studies on the sewage system also reported similar findings in the downstream water at the Ganges River, which showed a higher unique OTUs library compared to the upstream water (Reddy and Dubey, 2021). It is highly significant to associate the taxonomic richness observed in the WWTP microbial communities with ambient nitrogen and carbon availability within the sewage system (Johnson et al., 2015), biochemical presence (Numberger et al., 2019), and possible anthropogenic factors (Bojarczuk et al., 2018). The WHO’s One Health approach explains the spread and maintenance of resistance by analyzing all potentially involved ecosystems, including water bodies (Mackenzie and Jeggo, 2019). Interventions such as surveillance, control, and mitigation are carried out to combat bio-threats for the better health of individuals, populations, and the ecosystem. Conventional techniques such as ion exchange, carbon adsorption, evaporation, chemical precipitation, and membrane processes were the conventional methods of treating wastewater from domestic or industrial water (Vijayaraghavan et al., 2007; Wang and Chen, 2009; Narmadha and Kavitha, 2012; Shivajirao, 2012; Rajasulochana and Preethy, 2016). The current treatment uses the efficient green technology bioremediation method, which is a cost-effective and eco-friendly method, without generating by-products during pollutant degradation. The conventional methods of treating wastewater did not return the profitable price to performance ratio. The rapid growth of development aligned with exponential human population growth is deemed a time-saving and cost-effective method.

In the analysis of taxonomic composition profiles, only bacteria and archaea domains were showing statistically significant differences in mean value from influent to effluent. The bacteria domain was expected to show a reduction due to the overall aim of WWTPs to remove the pathogenic host, while the gradual archaea abundance was suggested to occur due to consensus of the non-pathogenic nature of archaea as primitive microorganisms and also been utilized in WWTPs as a collaborative partner of bacteria to remove pathogens during bioremediation (Aminov, 2013; Krzmarzick et al., 2018). Meanwhile, we suggested the non-significant difference value increase seen in the virus and eukaryote domains observed from influent to effluent water is due to a slight variation in the filtration and/or disinfection performance of different WWTP locations. Microfiltration for eukaryotic organisms and ultrafiltration of viruses in the secondary treatment stage removes microorganisms in wastewater. Prior to discharge, the treated water undergoes further disinfection with ultraviolet (UV) rays to deactivate unremoved microorganisms, especially for the domains with protective structural layers such as capsids of viruses. Double-stranded viruses, such as retrovirus and reovirus, exhibited high resistance capacity toward sedimentation or inactivation throughout the whole WWTP treatment process (Chahal et al., 2016; Corpuz et al., 2020). The remaining microorganisms of viruses and large-sized eukaryotes can multiply, grow, and populate the treated water even after being discharged. At the phylum-level taxa profile, *Proteobacteria*, *Bacteroidetes*, and *Firmicutes* were reported as the most abundant phyla in influent samples. *Bacteroidetes* and *Firmicutes* were reported as common human gut flora (Eckburg, 2005). However, *Firmicutes* were treated in the bioreactor and replaced with *Actinobacteria*, which showed an upward shift in the effluent samples. Analytical results from previous studies reported that *Proteobacteria* was also the most dominant phylum within the bacterial communities in soil (Roesch et al., 2007) and sewage (Oksanen et al., 2008; McLellan et al., 2010; Guo et al., 2015; Nascimento et al., 2018; Yasir, 2020). This finding supports the notion that the study of the bacterial composition of sewage provides a basis for the understanding of bacterial composition from human activities (Su et al., 2017). All of the top four phyla are curated in the Human Oral Microbiome Database and are significantly associated with illnesses such as asthma, gastrointestinal tract disease, and urinary tract infections (Rizzatti et al., 2017). Our analysis of the microbial composition profile of the other taxonomic levels displays the same decreasing pattern. Interestingly, species-wise, all six highly virulent pathogens species—*Enterococcus faecium*, *Staphylococcus aureus*, *Klebsiella pneumoniae*, *Acinetobacter baumannii*, *Pseudomonas aeruginosa*, and *Enterobacter* spp. (ESKAPE)—decreased from influent to effluent. The identified pathogens with a priority of ARG in all three categories also exhibited WWTP efficiency to control human pathogenic risks; the critical category (*E. coli*, *Serratia* spp., *Proteus* spp., *Morganella* spp.), high category (*Helicobacter pylori*, *Salmonella* spp.), and medium category (*Streptococcus pneumoniae*) were all observed to show a downward shift in the discharge water, albeit with a small relative mean difference (Asokan et al., 2019). Furthermore, the identified ARGs from our samples showed a similar reduction in the composition profile, indicating the WWTP filtering treatment stages function well and are moderately efficient. Despite not fully reducing these priority pathogens to a total of 0%, the low density of these opportunistic hosts is sufficient to control the bacterial quorum-sensing activities and improve the risk of water-borne infections (Zhang and Li, 2016; Maddela et al., 2019; Sivasankar et al., 2019). With the reduction of most opportunistic hosts within the sludge of WWTP in the effluent water, the occurrence of a significant reduction in abundance value of ARG in the effluent samples is expected. Despite being observed to have a richer microorganism profile, the pathogenicity of the effluent site showed less activity compared to the influent group (Figure 10). This shows that WWTP efficiency is not characterized by the total removal of microorganisms but through the performance of eliminating the opportunistic microorganisms carrying pathogenic genetic materials.

Correlation networks analysis is one of the most commonly applied methods to oversee patterns in the highly populated taxonomic group (Forsberg et al., 2012). The generated meta-network has been demonstrated as an applicable approach to predict associations of ARGs-ARGs and ARGs-species in both influent and effluent environments. Patterns of co-occurrence between nodes can show distinct niches, which may further explain the favorable conditions of certain species toward enrichment and survival (Freilich et al., 2010). In this study, only positive correlation Spearman’s ρ rho-value > 0.8 cut-offs were included. According to Faust and Raes (2012), positive correlations between nodes of a network may imply co-occurrence, co-colonization, and/or co-aggregation. According to the observation of effluent group meta-network, we suggested that effluent sites may have more diversified niches with high habitat heterogeneity (Olesen et al., 2007). As observed, it is worth highlighting the presence of *E. coli* as one of the main nodes with the most interaction within the network in both networks. Previously, *E. coli* have been reported several times in being poorly removed in WWTP (Anastasi et al., 2012). Although WWTPs were designed to remove *E. coli via* the biological and physicochemical methods in WWTPs (Frigon et al., 2013), the evidence of this intestinal pathogen persisted abundance in the environment near the effluent discharges sites raise concerns that need to be solved soon. Next, the co-occurrence network of influent and effluent water environment also listed a different module structure, which reveals those different environmental conditions play a big role in population interactions and associations. An application based on this finding may lead to a future study toward the pathogenicity of the species and their involvement in an inefficient WWTP treatment setting. In the sub-global networks, the three identified species from influent and effluent explicit meta-networks were from the *Gammaproteobacteria* class and *Proteobacteria* phyla. Despite not being listed as a category of pathogens for either ESKAPE or the WHO, *Enterobacteriales*, *A. sobria*, and *S. marcescens* may display a higher association with acquiring highly pathogenic ARGs than other species hosts.

Previously, PCR has been a conventional method for obtaining ecological data, such as the microbial profile, enabling the analysis of the total microbial communities present and its genetic potential within the environment. Yet, this technique’s major disadvantage is that it requires sequence information of the specific target gene of interest, as “unknown” taxons are inevitably limited to be analyzed using PCR. Hence, employing base sequences studies such as NGS, the shotgun metagenomics method is the recommended method in the present microbial ecologist’s toolbox as sequencing lays out big data information to be analyzed computationally, eliminating laborious workbench protocols. The introduction of metagenomics approaches allows for PCR-independent assessment for molecular investigation of biological activities within a community (Shah et al., 2010). Currently, studies on the pathogenic composition of wastewater are extended toward the investigation of potential horizontal or lateral gene transfer acquired between species or hosts (Jiao et al., 2017; Fouz et al., 2020; Nguyen et al., 2021). The nature of the wastewater treatment with a high concentration of bacterial communities increases the possibility of acting as a reservoir for gene transfer for some time. Such acquisition between species may occur as a response to the harsh and less-than-ideal internal environment of the wastewater treatment bioreactor for survival. Analyzing the intrinsic and extrinsic resistomes from our sample would provide a deeper understanding to confirm or dispute the hypothesis that a wastewater bioreactor could act as a reservoir for a gene-acquired process to happen as there is a risk of changing ARG distribution patterns (Aarestrup, 2015). The current study would also benefit from extending the analysis into the identification of contigs representing the plasmid (Eberhard, 1990) and protein-coding of mobile gene elements (MGEs), which are key vectors in assisting the horizontal gene transfer (HGT) between species. Employment of the PLSDB (Galata et al., 2019) and MGEfinder (Durrant et al., 2020) to retrieve information of plasmid and MGE present in current contigs, to evaluate the potential of HGT occurring in the municipal WWTP bioreactor. As of now, it is still difficult to determine the functions and roles of the identified microbial taxa in the bioreactor, either as key players in bioremediation or minor taxa. Furthermore, the interaction between bacteria or community in the WWTP bioreactor, irrespective of the treatment stage, remains elusive. The sequence of contigs retrieved from this study would allow us to further address the bioremediation technology, specifically in the annotation of the role the taxa plays (i.e., aerobic activation or anaerobic sludge) in the sanitation of wastewater (Zhang et al., 2012). Understanding the potential HGT risk will act as the first step in combating bacterial pathogens and delay the development of resistance (Aarestrup, 2015). The extracted information on associations retrieved from the co-occurrence meta-networks could be further utilized to obtain an in-depth understanding of the interactions between and within microbial populations but only with correct approaches to analysis (Williams et al., 2014; Freilich et al., 2018). Next, despite being able to perform the analysis of meta-network, we did not investigate the correlation of the presence of microbial taxa composition profile with any environmental factors such as temperature pH, depth of bioreactor ponds, presence of metal particles pollutants, and more. The study of metal-resistance genes (MRGs) together with our findings or ARGs in this study will provide further understanding and better interpretation toward microbial composition and the potential emergence of resistance genes as both antibiotic and metal provide a drive for HGT and natural selection to take place (Reddy and Dubey, 2019).

Our bioinformatics pipelines used high-performance tools that produce results with high accuracy and sensitivity. Gene noise cancellation was performed to ensure that the refined data are human genome free by using the BWA software. The NR catalog of ORF contigs was used to omit the possibility of analyzing overestimated data. The RGI used to align a constructed contigs library against CARD establishes deep enrichment of ARG information and sequence concentration. However, some novel types of ARGs in the NR catalog library might be missed as the RGI aligns to the database based on the similarity search that could lead to the underestimation of data. Still, some limitations could be addressed to further understand the effect of wastewater treatment technology in the filtering of pathogenic microbes. A validation test could be employed to test the presence of significant taxa of interest and thus confirm that data were retrieved from our *in vivo* metagenomics, which would conform to the extent and enhance the confidence of the results discussed.

## Conclusion

This research provides the basis for understanding the activity of the microbiome and its interaction within a specific environment. There is a global awareness among microbiologists toward the threat of potential emerging pathogenic bacteria with new genetic materials. Hence, the relevancy of this metagenomics study to be carried out is that it will contribute to filling the knowledge gaps in the microbial profile structure of the influent and effluent environment and the expected interactions between species depending on the environment. This study has successfully reported and exhibited solid evidence of the influent and effluent water profiles of microbial composition, ARGs, and the potential ARG–species associations in a national-scale analysis. Overall, all influent and effluent wastewater samples exhibit highly similar values in terms of their taxa composition profile patterns, indicating an efficient filtering and digestion system. The outcome of the study has met the health and environmental protection requirement such that the municipal WWTP sanitations have effectively minimized health risks, especially those stemming from water-borne infectious diseases, and environmental concerns of pathogen-contaminated effluent and influent water.

## Data Availability Statement

The datasets presented in this study can be found in online repositories. The names of the repository/repositories and accession number(s) can be found in the article/Supplementary Material.

## Author Contributions

MR collected the wastewater treatment plant samples at the influent and influent sources from all six sites. BA reported the raw read sequence to the NCBI BioProject, wrote the manuscript, performed the metagenomics analysis using the bioinformatics pipelines, and generated the figures and tables. NM guided the sampling location strategies and protocols, IN provided the metagenomics methodologies guidance for this studies, while FA guided in the generation and interpretation of the co-occurence networks. AO and IN contributed equally by proofreading the manuscript. All authors contributed to the article and approved the submitted version.

## Conflict of Interest

The authors declare that the research was conducted in the absence of any commercial or financial relationships that could be construed as a potential conflict of interest.

## Publisher’s Note

All claims expressed in this article are solely those of the authors and do not necessarily represent those of their affiliated organizations, or those of the publisher, the editors and the reviewers. Any product that may be evaluated in this article, or claim that may be made by its manufacturer, is not guaranteed or endorsed by the publisher.

## Abbreviations

WWTP: wastewater treatment plant
ARG: antibiotic resistance gene
NR: non-redundant
CARD: Comprehensive Antibiotic Resistance Database
PCA: principal component analysis
NMDS: non-metric multidimensional scaling.

## References

[B1] Archis A. Nathanson J. A. (2010). *Wastewater Treatment, Encyclopaedia Britannica.* Available online at: https://www.britannica.com/technology/wastewater-treatment (accessed September 16, 2020).

[B2] Aarestrup F. M. (2015). The livestock reservoir for antimicrobial resistance: a personal view on changing patterns of risks, effects of interventions and the way forward. *Philos. Trans. R. Soc. B Biol. Sci.* 370:20140085. 10.1098/rstb.2014.0085 25918442 PMC4424434

[B3] Al-Gheethi A. A. Efaq A. N. Bala J. D. Norli I. Abdel-Monem M. O. Ab. Kadir M. O. (2018). Removal of pathogenic bacteria from sewage-treated effluent and biosolids for agricultural purposes. *Appl. Water Sci.* 8 74. 10.1007/s13201-018-0698-6

[B4] Alcock B. P. Raphenya A. R. Lau T. T. Y. Tsang K. K. Bouchard M. Edalatmand A. (2019). CARD 2020: antibiotic resistome surveillance with the comprehensive antibiotic resistance database. *Nucleic Acids Res.* 48 D517–D525. 10.1093/nar/gkz935 31665441 PMC7145624

[B5] Altschul S. (1997). Gapped BLAST and PSI-BLAST: a new generation of protein database search programs. *Nucleic Acids Res.* 25 3389–3402. 10.1093/nar/25.17.3389 9254694 PMC146917

[B6] Aminov R. I. (2013). Role of archaea in human disease. *Front. Cell. Infect. Microbiol.* 3:42. 10.3389/fcimb.2013.00042 23964350 PMC3741462

[B7] Anastasi E. M. Matthews B. Stratton H. M. Katouli M. (2012). Pathogenic *Escherichia coli* found in sewage treatment plants and environmental waters. *Appl. Environ. Microbiol.* 78 5536–5541. 10.1128/AEM.00657-12 22660714 PMC3406122

[B8] Asokan G. Ramadhan T. Ahmed E. Sanad H. (2019). WHO Global Priority Pathogens List: a Bibliometric Analysis of Medline-PubMed for Knowledge Mobilization to Infection Prevention and Control Practices in Bahrain. *Oman Med. J.* 34 184–193. 10.5001/omj.2019.37 31110624 PMC6505350

[B9] Barberán A. Bates S. T. Casamayor E. O. Fierer N. (2012). Using network analysis to explore co-occurrence patterns in soil microbial communities. *ISME J.* 6 343–351. 10.1038/ismej.2011.119 21900968 PMC3260507

[B10] Bastian M. Heymann S. Jacomy M. (2009). “Gephi: an open source software for exploring and manipulating networks,” in *Proceedings of the 3rd International Aaai Conference on Weblogs and Social Media (ICWSM’09)*, (San Jose, CA).

[B11] Bojarczuk A. Jelonkiewicz Ł Lenart-Boroń A. (2018). The effect of anthropogenic and natural factors on the prevalence of physicochemical parameters of water and bacterial water quality indicators along the river Białka, southern Poland. *Environ. Sci. Pollut. Res.* 25 10102–10114. 10.1007/s11356-018-1212-2 29383643 PMC5891572

[B12] Bolger A. M. Lohse M. Usadel B. (2014). Trimmomatic: a flexible trimmer for Illumina sequence data. *Bioinformatics* 30 2114–2120. 10.1093/bioinformatics/btu170 24695404 PMC4103590

[B13] De Caceres M. Legendre P. (2009). Associations between species and groups of sites: indices and statistical inference. *Ecology* 90 3566–3574. 10.1890/08-1823.1 20120823

[B14] Chaffron S. Rehrauer H. Pernthaler J. von Mering C. (2010). A global network of coexisting microbes from environmental and whole-genome sequence data. *Genome Res.* 20 947–959. 10.1101/gr.104521.109 20458099 PMC2892096

[B15] Chahal C. van den Akker B. Young F. Franco C. Blackbeard J. Monis P. (2016). Pathogen and particle associations in wastewater: significance and implications for treatment and disinfection processes. *Adv. Appl. Microbiol.* 2016 63–119. 10.1016/bs.aambs.2016.08.001 27926432 PMC7126130

[B16] Clarke K. R. Warwick R. M. (1994). Similarity-based testing for community pattern: the two-way layout with no replication. *Mar. Biol.* 118 167–176. 10.1007/BF00699231

[B17] Conway J. R. Lex A. Gehlenborg N. (2017). UpSetR: an R package for the visualization of intersecting sets and their properties. *Bioinformatics* 33 2938–2940. 10.1093/bioinformatics/btx364 28645171 PMC5870712

[B18] Corpuz M. V. A. Buonerba A. Vigliotta G. Zarra T. Ballesteros F. Campiglia P. (2020). Viruses in wastewater: occurrence, abundance and detection methods. *Sci. Total Environ.* 745:140910. 10.1016/j.scitotenv.2020.140910 32758747 PMC7368910

[B19] Durrant M. G. Li M. M. Siranosian B. A. Montgomery S. B. Bhatt A. S. (2020). A bioinformatic analysis of integrative mobile genetic elements highlights their role in bacterial adaptation. *Cell Host Microbe* 27 140–153.e9. 10.1016/j.chom.2019.10.022 31862382 PMC6952549

[B20] Eberhard W. G. (1990). Evolution in bacterial plasmids and levels of selection. *Q. Rev. Biol.* 65 3–22. 10.1086/416582 2186429

[B21] Eckburg P. B. (2005). Diversity of the human intestinal microbial flora. *Science* 308 1635–1638. 10.1126/science.1110591 15831718 PMC1395357

[B22] Faust K. Raes J. (2012). Microbial interactions: from networks to models. *Nat. Rev. Microbiol.* 10 538–550. 10.1038/nrmicro2832 22796884

[B23] Forsberg K. J. Reyes A. Wang B. Selleck E. M. Sommer M. O. A. Dantas G. (2012). The shared antibiotic resistome of soil bacteria and human pathogens. *Science* 337 1107–1111. 10.1126/science.1220761 22936781 PMC4070369

[B24] Fouz N. Pangesti K. N. A. Yasir M. Al-Malki A. L. Azhar E. I. Hill-Cawthorne G. A. (2020). The contribution of wastewater to the transmission of antimicrobial resistance in the environment: implications of mass gathering settings. *Trop. Med. Infect. Dis.* 5:33. 10.3390/tropicalmed5010033 32106595 PMC7157536

[B25] Freilich M. A. Wieters E. Broitman B. R. Marquet P. A. Navarrete S. A. (2018). Species co-occurrence networks: can they reveal trophic and non-trophic interactions in ecological communities?. *Ecology* 99 690–699. 10.1002/ecy.2142 29336480

[B26] Freilich S. Kreimer A. Meilijson I. Gophna U. Sharan R. Ruppin E. (2010). The large-scale organization of the bacterial network of ecological co-occurrence interactions. *Nucleic Acids Res.* 38 3857–3868. 10.1093/nar/gkq118 20194113 PMC2896517

[B27] Frigon D. Biswal B. K. Mazza A. Masson L. Gehr R. (2013). Biological and physicochemical wastewater treatment processes reduce the prevalence of virulent *Escherichia coli*. *Appl. Environ. Microbiol.* 79 835–844. 10.1128/AEM.02789-12 23160132 PMC3568565

[B28] Fu L. Niu B. Zhu Z. Wu S. Li W. (2012). CD-HIT: accelerated for clustering the next-generation sequencing data. *Bioinformatics* 28 3150–3152. 10.1093/bioinformatics/bts565 23060610 PMC3516142

[B29] Galata V. Fehlmann T. Backes C. Keller A. (2019). PLSDB: a resource of complete bacterial plasmids. *Nucleic Acids Res.* 47 D195–D202. 10.1093/nar/gky1050 30380090 PMC6323999

[B30] GitHub - jstjohn/SeqPrep (2021). *Tool for Stripping Adaptors and/or Merging Paired Reads With Overlap into Single Reads. (No Date).* Available online at: https://github.com/jstjohn/SeqPrep (accessed September 5, 2021).

[B31] Guazzaroni M.-E. Beloqui A. Golyshin P. N. Ferrer M. (2009). Metagenomics as a new technological tool to gain scientific knowledge. *World J. Microbiol. Biotechnol.* 25 945–954. 10.1007/s11274-009-9971-z

[B32] Guo J. Peng Y. Ni B.-J. Han X. Fan L. Yuan Z. (2015). Dissecting microbial community structure and methane-producing pathways of a full-scale anaerobic reactor digesting activated sludge from wastewater treatment by metagenomic sequencing. *Microb. Cell Fact.* 14:33. 10.1186/s12934-015-0218-4 25880314 PMC4381419

[B33] Hammer Ø Harper D. A. T. Ryan P. D. (2001). PAST: paleontological statistics software package for education and data analysis. *Palaeontol. Electron.* 4:9.

[B34] IBM (2019). *IBM SPSS Statistics for Windows.* Armonk, NY: IBM.

[B35] Jiao Y.-N. Chen H. Gao R.-X. Zhu Y.-G. Rensing C. (2017). Organic compounds stimulate horizontal transfer of antibiotic resistance genes in mixed wastewater treatment systems. *Chemosphere* 184 53–61. 10.1016/j.chemosphere.2017.05.149 28578196

[B36] Johnson D. R. Lee T. K. Park J. Fenner K. Helbling D. E. (2015). The functional and taxonomic richness of wastewater treatment plant microbial communities are associated with each other and with ambient nitrogen and carbon availability. *Environ. Microbiol.* 17 4851–4860. 10.1111/1462-2920.12429 24552172

[B37] Joshi N. Fass J. (2011). *Sickle: A Sliding-Window, Adaptive, Quality-Based Trimming Tool for FastQ Files [Software].* Available online at: https://github.com/najoshi/sickle (accessed March 28, 2019).

[B38] Ju F. Xia Y. Guo F. Wang Z. Zhang T. (2014). Taxonomic relatedness shapes bacterial assembly in activated sludge of globally distributed wastewater treatment plants. *Environ. Microbiol.* 16 2421–2432. 10.1111/1462-2920.12355 24329969

[B39] Krzmarzick M. J. Taylor D. K. Fu X. McCutchan A. L. (2018). Diversity and niche of archaea in bioremediation. *Archaea* 2018 1–17. 10.1155/2018/3194108 30254509 PMC6140281

[B40] Li H. Durbin R. (2010). Fast and accurate long-read alignment with Burrows-Wheeler transform. *Bioinformatics (Oxford, England)* 26 589–595. 10.1093/bioinformatics/btp698 20080505 PMC2828108

[B41] Li R. Li Y. Kristiansen K. Wang J. (2008). SOAP: short oligonucleotide alignment program. *Bioinformatics* 24 713–714. 10.1093/bioinformatics/btn025 18227114

[B42] Mackenzie J. S. Jeggo M. (2019). The one health approach—why is it so important?. *Trop. Med. Infect. Dis.* 4:88. 10.3390/tropicalmed4020088 31159338 PMC6630404

[B43] Maddela N. R. Sheng B. Yuan S. Zhou Z. Villamar-Torres R. Meng F. (2019). Roles of quorum sensing in biological wastewater treatment: a critical review. *Chemosphere* 221 616–629. 10.1016/j.chemosphere.2019.01.064 30665091

[B44] Mancabelli L. Milani C. Lugli G. A. Turroni F. Mangifesta M. Viappiani A. (2017). Unveiling the gut microbiota composition and functionality associated with constipation through metagenomic analyses. *Sci. Rep.* 7:9879. 10.1038/s41598-017-10663-w 28852182 PMC5575163

[B45] Mandakovic D. Rojas C. Maldonado J. Latorre M. Travisany D. Delage E. (2018). Structure and co-occurrence patterns in microbial communities under acute environmental stress reveal ecological factors fostering resilience. *Sci. Rep.* 8:5875. 10.1038/s41598-018-23931-0 29651160 PMC5897386

[B46] McLellan S. L. Huse S. M. Mueller-Spitz S. R. Andreishcheva E. N. Sogin M. L. (2010). Diversity and population structure of sewage-derived microorganisms in wastewater treatment plant influent. *Environ. Microbiol.* 12 378–392. 10.1111/j.1462-2920.2009.02075.x 19840106 PMC2868101

[B47] Narayanan C. M. Narayan V. (2019). Biological wastewater treatment and bioreactor design: a review. *Sustain. Environ. Res.* 29:33. 10.1186/s42834-019-0036-1

[B48] Narmadha D. Kavitha V. J. M. S. (2012). Treatment of domestic waste water using natural flocculants. *Environ. Sci.* 7 173–178. 10.1016/0025-326x(83)90318-1

[B49] Nascimento A. L. Souza A. J. Andrade P. A. M. Andreote F. D. Coscione A. R. Oliveira F. C. (2018). Sewage sludge microbial structures and relations to their sources, treatments, and chemical attributes. *Front. Microbiol.* 9:1462. 10.3389/fmicb.2018.01462 30018612 PMC6037839

[B50] Newman M. E. J. (2006). Modularity and community structure in networks. *Proc. Natl. Acad. Sci. U.S.A.* 103 8577–8582. 10.1073/pnas.0601602103 16723398 PMC1482622

[B51] Nguyen A. Q. Vu H. P. Nguyen L. N. Wang Q. Djordjevic S. P. Donner E. (2021). Monitoring antibiotic resistance genes in wastewater treatment: current strategies and future challenges. *Sci.Total Environ.* 783:146964. 10.1016/j.scitotenv.2021.146964 33866168

[B52] Noguchi H. Park J. Takagi T. (2006). MetaGene: prokaryotic gene finding from environmental genome shotgun sequences. *Nucleic Acids Res.* 34 5623–5630. 10.1093/nar/gkl723 17028096 PMC1636498

[B53] Numberger D. Ganzert L. Zoccarato L. Mühldorfer K. Sauer S. Grossart H.-P. (2019). Characterization of bacterial communities in wastewater with enhanced taxonomic resolution by full-length 16S rRNA sequencing. *Sci. Rep.* 9:9673. 10.1038/s41598-019-46015-z 31273307 PMC6609626

[B54] O’Brien E. Xagoraraki I. (2019). A water-focused one-health approach for early detection and prevention of viral outbreaks. *One Health* 7:100094. 10.1016/j.onehlt.2019.100094 31080867 PMC6501061

[B55] Oksanen J. Kindt R. Legendre P. OHara B. Simpson G. L. Solymos P. M. (2008). *The Vegan Package, Community Ecology Package, (January).* 190. Available online at: https://cran.r-project.org/web/packages/vegan/vegan.pdf (accessed March 2, 2021).

[B56] Olesen J. M. Bascompte J. Dupont Y. L. Jordano P. (2007). The modularity of pollination networks. *Proc. Natl. Acad. Sci. U.S.A.* 104 19891–19896. 10.1073/pnas.0706375104 18056808 PMC2148393

[B57] Osińska A. Korzeniewska E. Harnisz M. Niestȩpski S. Jachimowicz P. (2019). The occurrence of antibiotic-resistant bacteria, including *Escherichia coli*, in Municipal Wastewater and River Water. *E3S Web Conf.* 100:00061. 10.1051/e3sconf/201910000061

[B58] R Core Team (2021). *R: A Language and Environment for Statistical Computing.* Vienna: R Core Team.

[B59] Rajasulochana P. Preethy V. (2016). Comparison on efficiency of various techniques in treatment of waste and sewage water – A comprehensive review. *Resour. Effic. Technol.* 2 175–184. 10.1016/j.reffit.2016.09.004

[B60] Reddy B. Dubey S. K. (2019). River Ganges water as reservoir of microbes with antibiotic and metal ion resistance genes: high throughput metagenomic approach. *Environ. Pollut.* 246 443–451. 10.1016/j.envpol.2018.12.022 30579213

[B61] Reddy B. Dubey S. K. (2021). Exploring the allochthonous pollution influence on bacterial community and co-occurrence dynamics of River Ganga water through 16S rRNA-tagged amplicon metagenome. *Environ. Sci. Pollut. Res.* 28 26990–27005. 10.1007/s11356-021-12342-w 33501578

[B62] Rizzatti G. Lopetuso L. R. Gibiino G. Binda C. Gasbarrini A. (2017). *Proteobacteria*: a Common Factor in Human Diseases. *BioMed Res. Int.* 2017 1–7. 10.1155/2017/9351507 29230419 PMC5688358

[B63] Roesch L. F. W. Fulthorpe R. R. Riva A. Casella G. Hadwin A. K. M. Kent A. D. (2007). Pyrosequencing enumerates and contrasts soil microbial diversity. *ISME J.* 1 283–290. 10.1038/ismej.2007.53 18043639 PMC2970868

[B64] Saito K. Koido S. Odamaki T. Kajihara M. Kato K. Horiuchi S. (2019). Metagenomic analyses of the gut microbiota associated with colorectal adenoma. *PLoS One* 14:e0212406. 10.1371/journal.pone.0212406 30794590 PMC6386391

[B65] Shah N. Tang H. Doak T. G. Ye Y. (2010). Comparing bacterial communities inferred from 16S rRNA gene sequencing and shotgun metagenomics. *Pac. Symp. Biocomput.* 2011 165–176. 10.1142/9789814335058_0018 21121044

[B66] Shikha, Singh S. Shankar S. (2021). “Microbial metagenomics,” in *Advances in Animal Genomics*, eds Mondal S. Singh R. L. (Amsterdam: Elsevier), 109–122. 10.1016/B978-0-12-820595-2.00008-4

[B67] Shivajirao P. A. (2012). Treatment of distillery wastewater using membrane technology. *Int. J. Adv. Eng. Res. Stud.* 1 275–283.

[B68] Sivasankar P. Poongodi S. Seedevi P. Sivakumar M. Murugan T. Loganathan S. (2019). Bioremediation of wastewater through a quorum sensing triggered MFC: a sustainable measure for waste to energy concept. *J. Environ. Manag.* 237 84–93. 10.1016/j.jenvman.2019.01.075 30780057

[B69] Spearman C. (1904). The proof and measurement of association between two things. *Am. J. Psychol.* 15:72. 10.2307/1412159 3322052

[B70] Von Sperling M. (2007). *Wastewater Characteristic, Treatment and Disposal. in Biological Wastewater Treatment Series.* London: IWA Publishing.

[B71] Su J.-Q. An X.-L. Li B. Chen Q.-L. Gillings M. R. Chen H. (2017). Metagenomics of urban sewage identifies an extensively shared antibiotic resistome in China. *Microbiome* 5:84. 10.1186/s40168-017-0298-y 28724443 PMC5517792

[B72] Vijayaraghavan K. Ahmad D. Ezani Bin Abdul Aziz M. (2007). Aerobic treatment of palm oil mill effluent. *J. Environ. Manag.* 82 24–31. 10.1016/j.jenvman.2005.11.016 16584834

[B73] Wang J. Chen C. (2009). Biosorbents for heavy metals removal and their future. *Biotechnol. Adv.* 27 195–226. 10.1016/j.biotechadv.2008.11.002 19103274

[B74] Ward A. J. Hobbs P. J. Holliman P. J. Jones D. L. (2008). Optimisation of the anaerobic digestion of agricultural resources. *Bioresour. Technol.* 99 7928–7940. 10.1016/j.biortech.2008.02.044 18406612

[B75] Wickham H. (2016). *ggplot2: Elegant Graphics for Data Analysis.* New York, NY: Springer-Verlag.

[B76] Wilcoxon F. (1945). Individual Comparisons by Ranking Methods. *Biomet. Bull.* 1:80. 10.2307/3001968

[B77] Williams R. J. Howe A. Hofmockel K. S. (2014). Demonstrating microbial co-occurrence pattern analyses within and between ecosystems. *Front. Microbiol.* 5:358. 10.3389/fmicb.2014.00358 25101065 PMC4102878

[B78] Wood D. E. Salzberg S. L. (2014). Kraken: ultrafast metagenomic sequence classification using exact alignments. *Genome Biol.* 15:R46. 10.1186/gb-2014-15-3-r46 24580807 PMC4053813

[B79] Xu H. Zhao F. Hou Q. Huang W. Liu Y. Zhang H. (2019). Metagenomic analysis revealed beneficial effects of probiotics in improving the composition and function of the gut microbiota in dogs with diarrhoea. *Food Funct.* 10 2618–2629. 10.1039/C9FO00087A 31021333

[B80] Yan L. (2021). *Package “ ggvenn “.*

[B81] Yasir M. (2020). Analysis of microbial communities and pathogen detection in domestic sewage using metagenomic sequencing. *Diversity* 13:6. 10.3390/d13010006

[B82] Zhang T. Shao M.-F. Ye L. (2012). 454 Pyrosequencing reveals bacterial diversity of activated sludge from 14 sewage treatment plants. *ISME J.* 6 1137–1147. 10.1038/ismej.2011.188 22170428 PMC3358032

[B83] Zhang W. Li C. (2016). Exploiting quorum sensing interfering strategies in gram-negative bacteria for the enhancement of environmental applications. *Front. Microbiol.* 6:1535. 10.3389/fmicb.2015.01535 26779175 PMC4705238

[B84] Zhang Z. Geng J. Tang X. Fan H. Xu J. Wen X. (2014). Spatial heterogeneity and co-occurrence patterns of human mucosal-associated intestinal microbiota. *ISME J.* 8 881–893. 10.1038/ismej.2013.185 24132077 PMC3960530

