# Supplementary material for: Metagenomics Insights Into the Microbial Diversity and Microbiome Network Analysis on the Heterogeneity of Influent to Effluent Water
Source: Front Microbiol. 2022 Apr 14;13:779196. doi: 10.3389/fmicb.2022.779196 (PMC9048743; doi:10.3389/fmicb.2022.779196)
Supplement: Supplementary file 1 [file Data_Sheet_1.docx]

Supplementary information of the article

Metagenomics insights into the microbial diversity and microbiome network analysis on influent to effluent water’s heterogeneity

Bahiyah Azli^1^, Mohd Nasharudin Razak^2^, Abdul Rahman Omar^1&3^, Nor Azimah Mohd Zain^4,5^, Fatimah Abdul Razak^6^, Nurulfiza Mat Isa^1&2*^

**SUPPLEMENTARY TABLE 1 Raw data**

**SUPPLEMENTARY TABLE 2: Clean data**

**SUPPLEMENTARY TABLE 3: Assembly statistic**

**SUPPLEMENTARY TABLE 4: Gene prediction statistic**

**SUPPLEMENTARY TABLE 5: Taxonomy profile**

**SUPPLEMENTARY TABLE 6: Alpha diversity metrics indices of microbial communities**

**SUPPLEMENTARY TABLE 7: Phylum information**

**SUPPLEMENTARY TABLE 8: Class information**

**SUPPLEMENTARY TABLE 9: Order information**

**SUPPLEMENTARY TABLE 10: Family information**

**SUPPLEMENTARY TABLE 11: Genus information**

**SUPPLEMENTARY TABLE 13: Phylum loading values (PCA and NMDS) and Scree plot (PCA), and further details of PCA’s plot**

**SUPPLEMENTARY TABLE 14: Phylum loading values (PCA and NMDS) and Scree plot (PCA), and further details of PCA’s plot**

**SUPPLEMENTARY TABLE 16: Resistance mechanism information**

**SUPPLEMENTARY TABLE 17: Drug class information**

**SUPPLEMENTARY TABLE 19: Topological properties of the co-occurrence gene meta-network**

**SUPPLEMENTARY TABLE 20:** **Geographical marking points of six chosen wastewater treatment plants as sampling stations in Johore, Malaysia of each location.**

| **#Sample_ID** | **Total_Reads** | **Total_Bases** | **Total_Reads_with_Ns** | **N_Reads%** | **N%** | **Error%** | **Q20%** | **Q30%** | **GC%** |
| --- | --- | --- | --- | --- | --- | --- | --- | --- | --- |
| JBT129E | 46632780.00 | 6994917000.00 | 11715904.00 | 25.12 | 0.29 | 0.03 | 96.08 | 90.96 | 54.30 |
| JBT129I | 48106944.00 | 7216041600.00 | 11957125.00 | 24.86 | 0.29 | 0.03 | 96.00 | 90.72 | 49.41 |
| JBT159E | 43311390.00 | 6496708500.00 | 14311.00 | 0.03 | 0.00 | 0.03 | 94.27 | 87.25 | 55.65 |
| JBT159I | 54374174.00 | 8156126100.00 | 18049.00 | 0.03 | 0.00 | 0.03 | 96.04 | 90.40 | 55.72 |
| JBT203E | 46700260.00 | 7005039000.00 | 7306567.00 | 15.65 | 0.18 | 0.03 | 96.46 | 91.60 | 52.93 |
| JBT203I | 56986472.00 | 8547970800.00 | 9084446.00 | 15.94 | 0.19 | 0.03 | 96.05 | 90.90 | 56.28 |
| JK1052E | 44927096.00 | 6739064400.00 | 10964185.00 | 24.40 | 0.29 | 0.03 | 96.20 | 91.16 | 56.14 |
| JK1052I | 44526700.00 | 6679005000.00 | 10868760.00 | 24.41 | 0.28 | 0.03 | 96.15 | 91.03 | 56.25 |
| JK1056E | 38049790.00 | 5707468500.00 | 8897273.00 | 23.38 | 0.27 | 0.03 | 95.97 | 90.76 | 54.48 |
| JK1056I | 62002268.00 | 9300340200.00 | 9857210.00 | 15.90 | 0.19 | 0.03 | 96.31 | 91.31 | 56.02 |
| JK1059E | 43073118.00 | 6460967700.00 | 14208.00 | 0.03 | 0.00 | 0.03 | 95.75 | 89.74 | 51.61 |
| JK1059I | 42772158.00 | 6415823700.00 | 14165.00 | 0.03 | 0.00 | 0.03 | 95.77 | 89.94 | 54.89 |

**SUPPLEMENTARY TABLE 1: Raw data**

| **#Sample_ID** | **Total_Reads** | **Total_Bases** | **Total_Reads_with_Ns** | **N_Reads%** | **N%** | **Error%** | **Q20%** | **Q30%** | **GC%** |
| --- | --- | --- | --- | --- | --- | --- | --- | --- | --- |
| JBT129E | 42979420.00 | 6409116463.00 | 10319083.00 | 24.01 | 0.25 | 0.03 | 97.74 | 93.37 | 54.08 |
| JBT129I | 44463468.00 | 6627716239.00 | 10511918.00 | 23.64 | 0.25 | 0.03 | 97.68 | 93.13 | 49.22 |
| JBT159E | 37626776.00 | 5599958640.00 | 12143.00 | 0.03 | 0.00 | 0.03 | 96.92 | 91.18 | 55.92 |
| JBT159I | 49981462.00 | 7457702498.00 | 16595.00 | 0.03 | 0.00 | 0.03 | 97.80 | 93.06 | 55.86 |
| JBT203E | 43271230.00 | 6457522614.00 | 6432171.00 | 14.86 | 0.16 | 0.03 | 97.96 | 93.78 | 52.76 |
| JBT203I | 52035666.00 | 7763156759.00 | 7914284.00 | 15.21 | 0.16 | 0.03 | 97.81 | 93.45 | 55.95 |
| JK1052E | 41566566.00 | 6198717763.00 | 9759356.00 | 23.48 | 0.25 | 0.03 | 97.77 | 93.48 | 56.05 |
| JK1052I | 41247408.00 | 6150425069.00 | 9632596.00 | 23.35 | 0.25 | 0.03 | 97.73 | 93.35 | 56.09 |
| JK1056E | 34773862.00 | 5184022091.00 | 7723693.00 | 22.21 | 0.24 | 0.03 | 97.72 | 93.30 | 54.10 |
| JK1056I | 57476890.00 | 8576604759.00 | 8660043.00 | 15.07 | 0.16 | 0.03 | 97.86 | 93.55 | 55.87 |
| JK1059E | 39452918.00 | 5882420783.00 | 12949.00 | 0.03 | 0.00 | 0.03 | 97.54 | 92.44 | 51.70 |
| JK1059I | 39059690.00 | 5825745851.00 | 12829.00 | 0.03 | 22.51 | 22.43 | 27.4 | 27.66 | 0 |

**SUPPLEMENTARY TABLE 2: Clean data**

| **#Sample_ID** | **ctg_num** | **ctg_length_all** | **ctg_n50** | **ctg_n90** | **ctg_max** | **ctg_min** |
| --- | --- | --- | --- | --- | --- | --- |
| JBT129E | 203586 | 292478272.00 | 1862 | 609 | 381945 | 500 |
| JBT129I | 260732 | 357372274.00 | 1696 | 600 | 589820 | 500 |
| JBT159E | 242497 | 311664421.00 | 1511 | 599 | 89057 | 500 |
| JBT159I | 252380 | 324802368.00 | 1533 | 601 | 192843 | 500 |
| JBT203E | 250644 | 308465190.00 | 1394 | 585 | 319803 | 500 |
| JBT203I | 142122 | 199587051.00 | 1803 | 597 | 356439 | 500 |
| JK1052E | 154412 | 239421814.00 | 2126 | 636 | 316717 | 500 |
| JK1052I | 153895 | 228596486.00 | 2079 | 614 | 331386 | 500 |
| JK1056E | 110539 | 194040642.00 | 2798 | 659 | 396996 | 500 |
| JK1056I | 224651 | 306619942.00 | 1709 | 612 | 308986 | 500 |
| JK1059E | 225149 | 312971219.00 | 1742 | 603 | 619501 | 500 |
| JK1059I | 173450 | 225019662.00 | 1558 | 597 | 354591 | 500 |

**SUPPLEMENTARY TABLE 3: Assembly statistic**

| **#Sample_ID** | **Source** | **ORFs** | **Total length(bp)** | **Average length(bp)** | **Max(bp)** | **Min(bp)** |
| --- | --- | --- | --- | --- | --- | --- |
| JBT129E | Effluent | 479270.00 | 270920250.00 | 565.28 | 30252.00 | 60.00 |
| JBT159E | Effluent | 512544.00 | 289895259.00 | 565.60 | 48177.00 | 60.00 |
| JBT203E | Effluent | 549163.00 | 288008513.00 | 524.45 | 23347.00 | 60.00 |
| JK1052E | Effluent | 371437.00 | 218373536.00 | 587.92 | 19145.00 | 60.00 |
| JK1056E | Effluent | 310326.00 | 178140248.00 | 574.04 | 63361.00 | 60.00 |
| JK1059E | Effluent | 512732.00 | 292647445.00 | 570.76 | 48317.00 | 60.00 |
| JBT129I | Influent | 518427.00 | 323654819.00 | 624.30 | 17310.00 | 60.00 |
| JBT159I | Influent | 491452.00 | 292201667.00 | 594.57 | 12813.00 | 60.00 |
| JBT203I | Influent | 295038.00 | 180456013.00 | 611.64 | 20618.00 | 60.00 |
| JK1052I | Influent | 331400.00 | 207048059.00 | 624.77 | 14292.00 | 60.00 |
| JK1056I | Influent | 457305.00 | 276320166.00 | 604.24 | 16556.00 | 60.00 |
| JK1059I | Influent | 339465.00 | 201228231.00 | 592.78 | 12484.00 | 60.00 |

**SUPPLEMENTARY TABLE 4: Gene prediction statistic**

| **#Sample_ID** | **Eukaryota** | **Viruses** | **Archaea** | **Bacteria** | **Other** |
| --- | --- | --- | --- | --- | --- |
| JBT129E | 118164 | 26284 | 18941 | 3960040 | 17366281 |
| JBT129I | 110447 | 10096 | 19121 | 10697664 | 11394406 |
| JBT159E | 104209 | 9178 | 14119 | 5042231 | 13643651 |
| JBT159I | 53029 | 34644 | 5391 | 17167471 | 7730196 |
| JBT203E | 136151 | 16195 | 15641 | 3118538 | 18349090 |
| JBT203I | 71863 | 11502 | 9853 | 16905174 | 9019441 |
| JK1052E | 78768 | 8576 | 10542 | 8992117 | 11693280 |
| JK1052I | 43320 | 7438 | 6819 | 15177702 | 5388425 |
| JK1056E | 98028 | 12509 | 13800 | 1123796 | 16138798 |
| JK1056I | 64776 | 43203 | 7269 | 19079763 | 9543434 |
| JK1059E | 130749 | 22560 | 12697 | 4153432 | 15407021 |
| JK1059I | 43254 | 4920 | 7328 | 13912187 | 5562156 |

**SUPPLEMENTARY TABLE 5: Taxonomy profile**

| **#Sample_ID** | **Source** | **Richness** | **Abundance** | **Shannon** | **Simpson** |
| --- | --- | --- | --- | --- | --- |
| JBT129I | Influent | 8656 | 607875 | 5.50878 | 0.984144 |
| JBT159I | Influent | 6718 | 130078.9 | 5.585815 | 0.988108 |
| JBT203I | Influent | 7336 | 487843.9 | 4.837649 | 0.970231 |
| JK1052I | Influent | 8183 | 152330.3 | 5.455508 | 0.982244 |
| JK1056I | Influent | 9817 | 155768.6 | 5.40651 | 0.975671 |
| JK1059I | Influent | 6221 | 206720 | 5.135428 | 0.976006 |
| JBT129E | Effluent | 10976 | 347360.7 | 6.373595 | 0.990678 |
| JBT159E | Effluent | 10717 | 528948.9 | 6.30796 | 0.99069 |
| JBT203E | Effluent | 10569 | 272373.4 | 6.507818 | 0.993121 |
| JK1052E | Effluent | 9690 | 159219.1 | 6.02119 | 0.982963 |
| JK1056E | Effluent | 9514 | 159519 | 7.259312 | 0.997707 |
| JK1059E | Effluent | 11052 | 411648.7 | 6.564773 | 0.99449 |

**SUPPLEMENTARY TABLE 6: Alpha diversity metrics indices of microbial communities**

| **Name Phylum** | **Influent Mean** | **Effluent Mean** | **Mean diff** | **%** |
| --- | --- | --- | --- | --- |
| Proteobacteria | 775698 | 258039.7 | 517658.3 | 67% |
| Bacteroidetes | 103767.6 | 22715.2 | 81052.36 | 78% |
| Firmicutes | 8659.997 | 3615.105 | 5044.892 | 58% |
| Actinobacteria | 5683.992 | 88007.07 | -82323.1 | -1448% |
| Viruses_norank | 3837.331 | 10693.21 | -6855.88 | -179% |
| Bacteria_norank | 1517.079 | 5573.022 | -4055.94 | -267% |
| Verrucomicrobia | 611.1113 | 11238.59 | -10627.5 | -1739% |
| Others | 142.6007 | 7861.963 | -7719.36 | -5413% |
| Planctomycetes | 142.6007 | 7861.963 | -7719.36 | -5413% |

**SUPPLEMENTARY TABLE 7: Phylum information**

| **Name Class** | **Influent Mean** | **Effluent Mean** | **Mean diff** | **%** |
| --- | --- | --- | --- | --- |
| Gammaproteobacteria | 606321.8 | 94371.72 | 511950.1 | 84% |
| Betaproteobacteria | 153817.5 | 123944.7 | 29872.76 | 19% |
| Flavobacteriia | 50894 | 8302.211 | 42591.79 | 84% |
| Bacteroidia | 48753.19 | 3945.385 | 44807.8 | 92% |
| Others | 36114.42 | 86295.04 | -50180.6 | -139% |
| Alphaproteobacteria | 4604.712 | 35395.94 | -30791.2 | -669% |
| Actinobacteria | 4037.328 | 78247.85 | -74210.5 | -1838% |
| Viruses_norank | 3837.331 | 10693.21 | -6855.88 | -179% |

**SUPPLEMENTARY TABLE 8: Class information**

| **Name Order** | **Influent Mean** | **Effluent Mean** | **Mean diff** | **%** |
| --- | --- | --- | --- | --- |
| Pseudomonadales | 501992.6 | 76121.91 | 425870.7 | 85% |
| Burkholderiales | 119643.1 | 64272.68 | 55370.44 | 46% |
| Others | 58076.48 | 156661.3 | -98584.8 | -170% |
| Flavobacteriales | 48948.33 | 7751.978 | 41196.35 | 84% |
| Bacteroidales | 47940.91 | 3585.15 | 44355.76 | 93% |
| Aeromonadales | 45086.78 | 2463.542 | 42623.24 | 95% |
| Alteromonadales | 35648.55 | 1976.809 | 33671.74 | 94% |
| Rhodocyclales | 13110.93 | 31961.62 | -18850.7 | -144% |
| Enterobacterales | 13099.01 | 1665.767 | 11433.24 | 87% |
| Neisseriales | 11681.49 | 4770.87 | 6910.619 | 59% |
| Nitrosomonadales | 6417.918 | 10725.01 | -4307.1 | -67% |
| Betaproteobacteria_norank | 2858.636 | 11962.71 | -9104.08 | -318% |
| Rhizobiales | 2208.051 | 14569.65 | -12361.6 | -560% |
| Micrococcales | 921.5366 | 19851.29 | -18929.7 | -2054% |
| Corynebacteriales | 459.4096 | 14231.6 | -13772.2 | -2998% |
| Actinobacteria_norank | 286.5278 | 18624.22 | -18337.7 | -6400% |

**SUPPLEMENTARY TABLE 9: Order information**

| **Name Family** | **Influent Mean** | **Effluent Mean** | **Mean diff** | **%** |
| --- | --- | --- | --- | --- |
| Pseudomonadaceae | 498880.3 | 75497.35 | 423383 | 85% |
| Others | 141485.4 | 228167.9 | -86682.4 | -61% |
| Oxalobacteraceae | 70597.92 | 9917.829 | 60680.09 | 86% |
| Flavobacteriaceae | 46758.22 | 5729.308 | 41028.91 | 88% |
| Aeromonadaceae | 44620.88 | 2453.471 | 42167.41 | 95% |
| Comamonadaceae | 38906.6 | 25401.23 | 13505.37 | 35% |
| Shewanellaceae | 34744.35 | 938.6142 | 33805.73 | 97% |
| Bacteroidaceae | 14448.91 | 1407.112 | 13041.79 | 90% |
| Azonexaceae | 6748.811 | 7370.367 | -621.556 | -9% |
| Burkholderiaceae | 3694.169 | 18445.93 | -14751.8 | -399% |
| Rhodocyclaceae | 3547.747 | 18242.07 | -14694.3 | -414% |
| Betaproteobacteria_norank | 2858.636 | 11962.71 | -9104.08 | -318% |
| Microbacteriaceae | 801.7613 | 17037.98 | -16236.2 | -2025% |
| Actinobacteria_norank | 286.5278 | 18624.22 | -18337.7 | -6400% |

**SUPPLEMENTARY TABLE 10: Family information**

| **Name Genus** | **Influent Mean** | **Effluent Mean** | **Mean diff** | **%** |
| --- | --- | --- | --- | --- |
| Pseudomonas | 498678.1 | 75400.79 | 423277.3 | 85% |
| Others | 152712.8 | 241859.7 | -89146.9 | -58% |
| Janthinobacterium | 63561.23 | 7181.916 | 56379.31 | 89% |
| Shewanella | 34744.35 | 938.6142 | 33805.73 | 97% |
| Flavobacterium | 34074.86 | 3856.823 | 30218.04 | 89% |
| Aeromonas | 26840.54 | 1230.783 | 25609.76 | 95% |
| Acidovorax | 20529.6 | 3539.83 | 16989.77 | 83% |
| Tolumonas | 17389.32 | 1176.601 | 16212.72 | 93% |
| Bacteroides | 14401.59 | 1404.312 | 12997.28 | 90% |
| Prevotella | 12124.43 | 412.4347 | 11712 | 97% |
| Rivicola | 8574.251 | 2042.736 | 6531.515 | 76% |
| Chryseobacterium | 6883.721 | 288.89 | 6594.831 | 96% |
| Comamonadaceae_norank | 6072.481 | 1968.214 | 4104.267 | 68% |
| Dechloromonas | 6003.655 | 5437.622 | 566.0329 | 9% |
| Bacteria_norank | 1511.046 | 5553.578 | -4042.53 | -268% |
| Betaproteobacteria_norank | 1477.897 | 7113.532 | -5635.63 | -381% |
| Polynucleobacter | 812.4822 | 12249.48 | -11437 | -1408% |
| Azospira | 499.0871 | 6318.544 | -5819.46 | -1166% |
| Limnohabitans | 459.801 | 11785.68 | -11325.9 | -2463% |
| Actinobacteria_norank | 285.4138 | 18597.1 | -18311.7 | -6416% |
| Aurantimicrobium | 250.4848 | 8877.322 | -8626.84 | -3444% |
| Rhodocyclaceae_norank | 248.8852 | 6540.024 | -6291.14 | -2528% |
| Mycobacterium | 177.3043 | 7442.784 | -7265.48 | -4098% |
| Candidatus Planktophila | 67.05111 | 9978.738 | -9911.69 | -14782% |

**SUPPLEMENTARY TABLE 11: Genus information**

| **#Sample_ID** | **PCA** | | | | | | | | | | **NMDS** | |
| --- | --- | --- | --- | --- | --- | --- | --- | --- | --- | --- | --- | --- |
|  | **PC 1** | **PC 2** | **PC 3** | **PC 4** | **PC 5** | **PC 6** | **PC 7** | **PC 8** | **PC 9** | **PC 10** | **Axis 1** | **Axis 2** |
| JBT129E | -2.6188E05 | -23921 | 1633.6 | -12370 | -12142 | -5556.3 | 1462.7 | 343.69 | -43.757 | 10.884 | -0.20074 | -0.11276 |
| JBT159E | -2.1989E05 | -52654 | 1.0489E05 | 11060 | -5793 | 3739.2 | 646.32 | -1107.3 | 181.81 | 22.155 | -0.1825 | -0.15014 |
| JBT203E | -3.736E05 | -24053 | 19343 | -11249 | 7011.7 | 8861.7 | -2126.8 | 340 | 71.556 | 26.805 | -0.40086 | -0.14328 |
| JK1052E | -35421 | -28160 | -49841 | 3292.9 | -1981.2 | -3717.1 | -5421.4 | -2619.3 | 627.16 | -8.8008 | 0.044819 | -0.066391 |
| JK1056E | -4.4051E05 | -12366 | -1.0544E05 | 8324.1 | 202.69 | 2839.2 | 2787 | 648.71 | -163.46 | 37.231 | -0.38522 | 0.41539 |
| JK1059E | -2.5358E05 | -10533 | 41394 | 2525.5 | 10556 | -10429 | 74.105 | 1388.7 | -252.53 | 6.5142 | -0.17903 | -0.124 |
| JBT129I | 21897 | 2.8101E05 | 11347 | 232.69 | -475.44 | 597.97 | 334.2 | -583.43 | 155.05 | 20.456 | 0.24329 | -0.16185 |
| JBT159I | 3.256E05 | -38183 | -5839 | -3816.4 | 5053.9 | -336.77 | 3207.4 | -2517 | 939.71 | 763.17 | 0.2082 | 0.074357 |
| JBT203I | 2.6391E05 | 8333.9 | -5504.8 | 963.83 | -1509.7 | 999.5 | -1567.2 | 844.41 | -1809.5 | -404.33 | 0.21432 | 0.057898 |
| JK1052I | 3.3965E05 | -31511 | -3766.9 | 1251.8 | -1652.5 | 1248 | -725.56 | 1594.5 | -733.53 | 1602.5 | 0.21774 | 0.072059 |
| JK1056I | 3.0481E05 | -49486 | -3862.8 | -1755.9 | 2204.2 | 638.37 | 1813.8 | -1574.8 | -854.12 | -1330.1 | 0.20382 | 0.070301 |
| JK1059I | 3.2902E05 | -18475 | -4359 | 1540.7 | -1474.9 | 1114.8 | -484.5 | 3241.7 | 1881.6 | -746.55 | 0.21617 | 0.068418 |


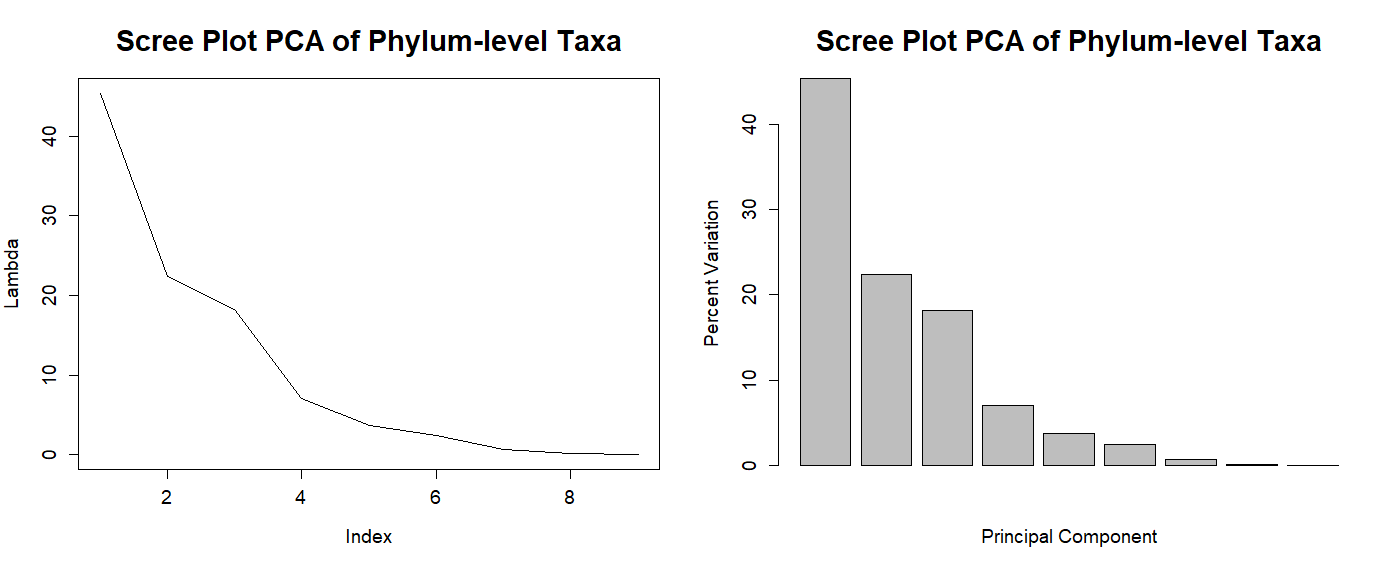


| **Phyla** | **Rotation value** |
| --- | --- |
| Proteobacteria | -0.1883777 |
| Verrucomicrobia | 0.4541283 |
| Actinobacteria | 0.4434652 |
| Viruses_norank | 0.2661370 |
| Planctomycetes | 0.6290094 |
| Bacteroidetes | -0.1736267 |
| Others | 0.1661449 |
| Bacteria_norank | 0.1440706 |
| Firmicutes | -0.1289631 |

| Proteobacteria | Viruses_norank  Actinobacteria  Planctomycetes  Verrucomicrobia |
| --- | --- |
| Bacteroidetes  Firmicutes | Others  Bacteria_norank |

*****Note: The text inside the 2 rows and 2 columns represent the variable text seen in Figure 5a.**

**SUPPLEMENTARY TABLE 13: Phylum loading values (PCA and NMDS) and Scree plot (PCA), and further details of PCA’s plot. Values were retrieved using SUPPLEMENTARY TABLE v.4.03.**

| **#Sample_ID** | **PCA** | | | | | | | | | | **NMDS** | |
| --- | --- | --- | --- | --- | --- | --- | --- | --- | --- | --- | --- | --- |
|  | **PC 1** | **PC 2** | **PC 3** | **PC 4** | **PC 5** | **PC 6** | **PC 7** | **PC 8** | **PC 9** | **PC 10** | **Axis 1** | **Axis 2** |
| JBT129E | -2.6093E05 | -18666 | 10034 | 3245.1 | -2069.6 | 2486.5 | -35651 | -12972 | -3255.3 | -615.43 | -0.32502 | -0.086624 |
| JBT159E | -2.9272E05 | -6362.9 | 33264 | 56388 | -12687 | -1584.7 | 20882 | -11351 | -2569.7 | -443.33 | -0.34674 | -0.085969 |
| JBT203E | -2.621E05 | -38294 | -16131 | -5672.2 | -2265 | 1192.6 | 1058.7 | 20226 | -16094 | -1506.2 | -0.33559 | -0.046495 |
| JK1052E | 62461 | -31125 | -47479 | -25632 | -9431.4 | -16514 | 5444.6 | -2257.6 | 4216.3 | -3774.8 | 0.19758 | 0.014184 |
| JK1056E | -2.8006E05 | -55975 | -48776 | -46951 | 10423 | 3335.4 | 13434 | -10518 | 2349.4 | 2312.1 | -0.19502 | 0.39366 |
| JK1059E | -2.5085E05 | -20875 | 7133 | 16041 | -8116.6 | 353.76 | -6068.7 | 15933 | 18103 | 1659.1 | -0.32161 | -0.071107 |
| JBT129I | -50004 | 1.3482E05 | 1.219E05 | -34837 | 4649.5 | -4095.1 | 3901.4 | 937.82 | -175.07 | 406.16 | 0.18141 | -0.071619 |
| JBT159I | 3.3924E05 | -48640 | 11714 | 673.45 | -11063 | 12027 | -57.013 | 45.343 | -2635.8 | 12885 | 0.25061 | 0.0010064 |
| JBT203I | 77315 | 1.7701E05 | -1.0341E05 | 14093 | 4486.3 | 12093 | -814.72 | 99.824 | -291.73 | 372.46 | 0.19073 | -0.031321 |
| JK1052I | 3.5158E05 | -47109 | 26401 | -4688.4 | -18097 | 30599 | 1841.7 | -793.49 | 1556.9 | -8776.2 | 0.2408 | -0.0053314 |
| JK1056I | 2.1443E05 | -44668 | 13313 | 19561 | 64915 | -4388.1 | -99.719 | 1020.7 | 730.6 | -1501.7 | 0.22442 | -0.00065187 |
| JK1059I | 3.5163E05 | -115.96 | -7957.1 | 7779.2 | -20744 | -35505 | -3871.3 | -370.56 | -1934.4 | -1017.5 | 0.23843 | -0.0097351 |

**
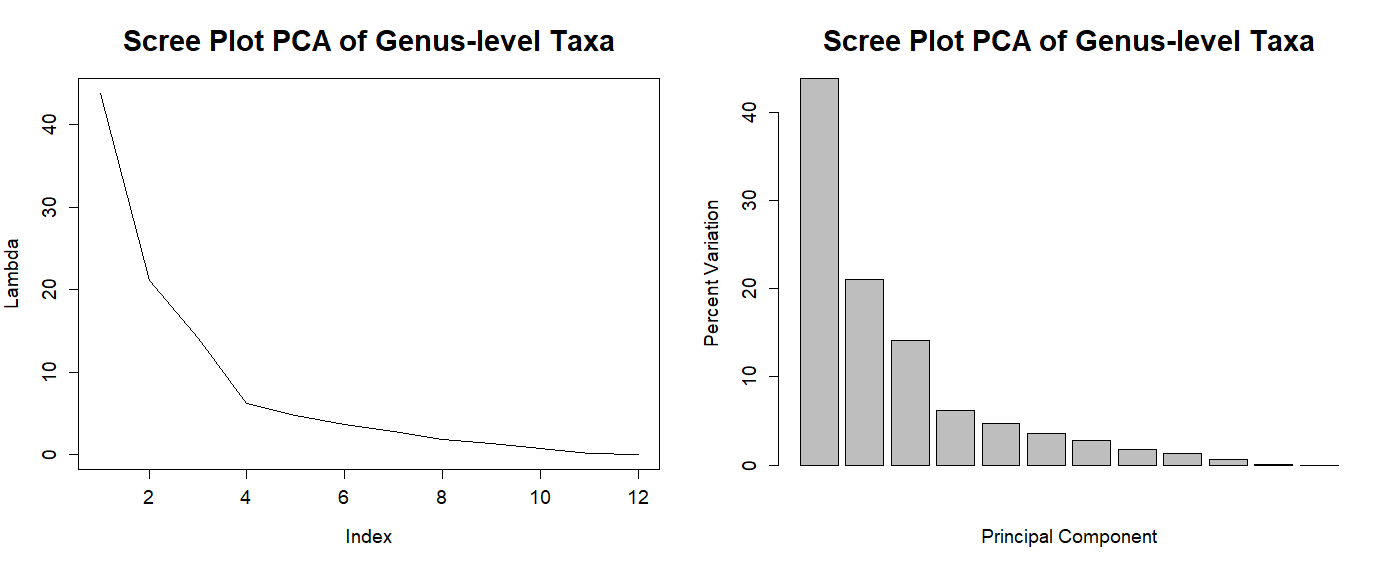
**

| **Genus** | **Ordination value** |
| --- | --- |
| Pseudomonas | -0.24204918 |
| Others | 0.24594390 |
| Janthinobacterium | -0.11986643 |
| Shewanella | -0.14664817 |
| Flavobacterium | -0.11779518 |
| Aeromonas | -0.14555409 |
| Acidovorax | -0.21583910 |
| Tolumonas | -0.19066958 |
| Bacteroides | -0.21697880 |
| Prevotella | -0.10997515 |
| Rivicola | -0.19237950 |
| Chryseobacterium | -0.11358262 |
| Comamonadaceae_norank | -0.17209517 |
| Dechloromonas | 0.09717361 |
| Bacteria_norank | 0.06013085 |
| Betaproteobacteria_norank | 0.27517580 |
| Polynucleobacter | 0.26976372 |
| Azospira | 0.25661482 |
| Limnohabitans | 0.27025067 |
| Actinobacteria_norank | 0.27636114 |
| Aurantimicrobium | 0.20081071 |
| Rhodocyclaceae_norank | 0.26033739 |
| Mycobacterium | 0.17429907 |
| Candidatus Planktophila | 0.27027713 |

| Shewanella  Aeromonas  Rivicola  Tolumonas  Pseudomonas | Bacteria_norank  Dechloromonas |
| --- | --- |
| Bacteroides  Acidovorax  Janthinobacterium  Comamonadaceae_norank  Chryseobacterium  Flavobacterium  Prevotella | Candidatus Planktophila  Limnohabitans  Polynucleobacter  Rhodocyclaceae_norank  Azospira  Aurantimicrobium  Mycobacterium  Others  Actinobacteria_norank  Betaproteobacteria_norank |

*****Note: The text inside the 2 rows and 2 columns represent the variable text seen in Figure 5b.**

**SUPPLEMENTARY TABLE 14: Genus loading values (PCA and NMDS) and Scree plot (PCA), and further details of PCA’s plot. Values were retrieved using SUPPLEMENTARY TABLE v.4.03.**

| **Resistance Mechanism** | **Influent Mean** | **Effluent Mean** | **Mean diff** | **%** |
| --- | --- | --- | --- | --- |
| reduced permeability to antibiotic;resistance by absence | 264.4661 | 31.68837 | -232.778 | 88% |
| antibiotic target alteration;antibiotic efflux;reduced permeability to antibiotic | 991.5178 | 151.005 | -840.513 | 85% |
| resistance by absence | 3148.345 | 506.7305 | -2641.61 | 84% |
| reduced permeability to antibiotic | 1699.147 | 302.591 | -1396.56 | 82% |
| antibiotic efflux;reduced permeability to antibiotic | 411.5102 | 74.3493 | -337.161 | 82% |
| antibiotic target alteration;antibiotic efflux | 1928.495 | 499.3209 | -1429.17 | 74% |
| antibiotic efflux | 48326.6 | 17237.82 | -31088.8 | 64% |
| antibiotic inactivation | 5021.14 | 2072.628 | -2948.51 | 59% |
| antibiotic target alteration | 26942.62 | 16744.22 | -10198.4 | 38% |
| antibiotic target replacement | 549.7681 | 442.282 | -107.486 | 20% |
| antibiotic target protection | 2441.044 | 2153.167 | -287.877 | 12% |
| antibiotic target alteration;antibiotic target replacement | 610.6751 | 690.4419 | 79.7668 | -13% |
| Resistance Mechanism | Influent | Effluent | Mean diff | % |
| reduced permeability to antibiotic;resistance by absence | 264.4661 | 31.68837 | -232.778 | 88% |
| antibiotic target alteration;antibiotic efflux;reduced permeability to antibiotic | 991.5178 | 151.005 | -840.513 | 85% |
| resistance by absence | 3148.345 | 506.7305 | -2641.61 | 84% |
| reduced permeability to antibiotic | 1699.147 | 302.591 | -1396.56 | 82% |
| antibiotic efflux;reduced permeability to antibiotic | 411.5102 | 74.3493 | -337.161 | 82% |
| antibiotic target alteration;antibiotic efflux | 1928.495 | 499.3209 | -1429.17 | 74% |

**SUPPLEMENTARY TABLE 16: Resistance mechanism information**

| **Drug Class** | **Influent Mean** | **Effluent Mean** | **Mean diff** | **%** |
| --- | --- | --- | --- | --- |
| Phenicol | 723.0974 | 149.0961 | -574.001 | 79% |
| Cephamycin | 78.24493 | 16.23835 | -62.0066 | 79% |
| Penam | 60.76315 | 14.73015 | -46.033 | 76% |
| Aminoglycoside | 4120.375 | 1216.992 | -2903.38 | 70% |
| MDR | 62056.64 | 23616.53 | -38440.1 | 62% |
| Lincosamide | 207.5323 | 85.36104 | -122.171 | 59% |
| Nitrofuran | 33.17997 | 13.78268 | -19.3973 | 58% |
| Triclosan | 60.18335 | 25.15248 | -35.0309 | 58% |
| Nucleoside | 287.1819 | 125.4711 | -161.711 | 56% |
| Fosfomycin | 1811.444 | 805.4138 | -1006.03 | 56% |
| Glycopeptide | 5996.243 | 2860.017 | -3136.23 | 52% |
| Cephalosporin | 116.7989 | 57.80196 | -58.9969 | 51% |
| Peptide | 8873.933 | 4946.319 | -3927.61 | 44% |
| MLS | 453.6841 | 289.1994 | -164.485 | 36% |
| Diaminopyrimidine | 265.409 | 175.3529 | -90.0562 | 34% |
| Rifamycin | 529.0012 | 373.0934 | -155.908 | 29% |
| Pyrazinamide | 718.3937 | 551.6598 | -166.734 | 23% |
| Isoniazid | 395.398 | 312.1567 | -83.2412 | 21% |
| Fusidic Acid | 957.2953 | 771.7879 | -185.507 | 19% |
| Streptogramin | 197.2258 | 161.561 | -35.6648 | 18% |
| Para-Aminosalicylic Acid | 650.5925 | 542.3248 | -108.268 | 17% |
| Tetracycline | 976.6197 | 827.8851 | -148.735 | 15% |
| Macrolide | 48.92589 | 42.27738 | -6.6485 | 14% |
| Elfamycin | 893.8615 | 830.7467 | -63.1148 | 7% |
| Mupirocin | 1344.953 | 1251.273 | -93.6795 | 7% |
| Aminocoumarin | 195.823 | 227.9771 | 32.15415 | -16% |
| Fluoroquinolone | 515.8734 | 629.4727 | 113.5993 | -22% |
| Carbapenem | 173.061 | 214.0484 | 40.98745 | -24% |
| Polyamine | 46.83402 | 58.39291 | 11.55889 | -25% |
| Sulfonamide | 0.436015 | 3.339933 | 2.903918 | -666% |

**SUPPLEMENTARY TABLE 17: Drug class**

| **Environment** | **Number of nodes** | **Number of edges** | **Modularity (MD)** | **Average clustering coefficient (CC)** | **Average path length (APL)** | **Network diameter (ND)** | **Average degree (AD)** | **Graph density** |
| --- | --- | --- | --- | --- | --- | --- | --- | --- |
| Influent | 1352 | 28025 | 0.683 | 0.563 | 3.122 | 8 | 41.457 | 0.031 |
| Effluent | 1352 | 33932 | 0.691 | 0.611 | 3.096 | 7 | 50.195 | 0.037 |
| Filtered influent | 212 | 2421 | 0.139 | 0.563 | 3.152 | 8 | 22.84 | 0.108 |
| Filtered effluent | 216 | 3960 | 0.195 | 0.746 | 3.826 | 11 | 36.667 | 0.171 |

**SUPPLEMENTARY TABLE 19: Topological properties of the co-occurrence meta-network of different environment settings**

| **Code** | **Latitude** | **Longitude** |
| --- | --- | --- |
| JBT129 | 1.502379901 | 103.6138547 |
| JBT159 | 1.53690635 | 103.6363106 |
| JBT203 | 1.511418344 | 103.6303277 |
| JK1052 | 1.60352948 | 103.6399832 |
| JK1056 | 1.5058317 | 103.6205309 |
| JK1059 | 1.633588541 | 103.6117179 |

**SUPPLEMENTARY TABLE 20: Geographical marking points of six chosen wastewater treatment plants as sampling stations in Johore, Malaysia of each location.**
